# Molten Salt Derived MXenes: Synthesis and Applications

**DOI:** 10.1002/advs.202307106

**Published:** 2024-07-17

**Authors:** Dawid D. Kruger, Hermenegildo García, Ana Primo

**Affiliations:** ^1^ Instituto Universitario de Tecnología Química CSIC‐UPV Universitat Politècnica de València Av. De los Naranjos s/n València 46022 Spain

**Keywords:** molten salt synthesis, mxene, surface modification, lewis acid max etchants

## Abstract

About one decade after the first report on MXenes, these 2D early transition metal carbides or nitrides have become among the best‐performing materials in electrode applications related to electrical energy storage devices and power‐to‐fuels conversion. MXenes are obtained by a top‐down approach starting from the appropriate 3D MAX phase that undergoes etching of the A‐site metal. Initial etching procedures are based on the use of concentrated HF or the in situ generation of this highly corrosive and poisonous reagent. Etching of the MAX phase is one of the major hurdles limiting the progress of the field. The present review summarizes an alternative, universal, and easily scalable etching procedure based on treating the MAX precursor with a Lewis acid molten salt. The review starts with presenting the current state of the art of the molten salt etching procedure to obtain or modify MXene, followed by a summary of the applications of these MXene samples. The aim of the review is to show the versatility and advantages of molten salt etching in terms of general applicability, control of the surface terminal groups, and uniform deposition of metal nanoparticles, among other features of the procedure.

## Graphical abstract

Synthesis methods of MXenes are rapidly advancing with the use of halide molten salt etchants. One‐step synthesis of MXene‐metal nanoparticle hybrids are enabled, and new areas of MXene surface chemistry are unlocked to include heavier halogens, and chalcogens by post‐synthetic substitution. Herein, molten salt synthesis of MXene and MXene‐hybrids and their applications in catalysis and energy storage are reviewed.

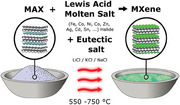

## 1 Introduction

Since the first report on MXene synthesis over one decade ago,^[^ ^1^ ^]^ these 2D nanomaterials have attracted considerable interest due to their numerous potential applications.^[^ ^2^ ^]^ MXenes are 2D nanomaterials constituted by alternating layers of one atom thick sheet of an early transition metal (denoted as *M*) with one atom thick sheet of carbide or nitride (denoted as *X*). The metal layers are always external, and for this reason, the general formula is M_n+1_X_n_, where *n* may be an integer number from 1 to 4. The external metal sheets generally have surface termination groups (denoted as *T*), that exert a considerable influence on the electronic properties, behavior, and performance of the MXene sample.^[^ ^3^, ^4^, ^5^ ^]^

**Figure 1:**
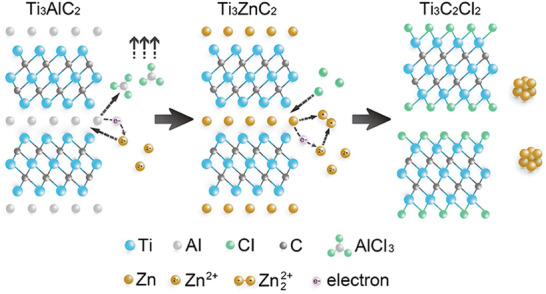
Illustration of the mechanism of the etching of MAX phase by Lewis acid molten (ZnCl_2_) salt via A‐site replacement followed by halide termination into multilayer MXene. Reproduced with permission.^[^ ^8^ ^]^ Copyright 2019, American Chemical Society.

MXenes are obtained from the corresponding M_n_AX_n‐1_ phase (generally denoted as MAX, the A‐site element being a main group or late transition metal, like Al, Si, Ga, Zn, Cu) by a top‐down approach. The metallic nature of the bond between the A metal and the MXene layers makes possible the selective etching of the A metal converting the 3D MAX solid into the 2D MXene. **Figure** **1** illustrates the process of MXene synthesis. In many cases, MAX precursors can be obtained by high‐temperature solid phase synthesis of the corresponding elements. There is considerable flexibility in the make‐up of MAX phases that can include ordered/disordered, multi‐metallic M and/or A sites, carbo‐nitrides and borides, variation in the number of layers *n*, and particle size and morphology, therefore, offering many possibilities in the structure and composition MAX phases.^[^ ^6^, ^7^ ^]^

One of the main bottlenecks in MXene synthesis is to determine the optimal conditions for the “A” metal etching. Initial reports on MXene synthesis described etching procedures using HF, with concentration, temperature, and treatment time being the main parameters to be optimized. Subsequent refinements of fluoride etching were the in situ synthesis of HF by reacting HCl or a strong acid with a fluoride‐containing salt, NH_4_F or NaBF_4_ being among the most common ones. This difficulty in finding and optimizing successful fluoride‐based etching methods determined that the initial studies on MXenes were dealing with only a few MXenes for which the preparation procedure was reliable. The list includes Ti_2_C, Nb_2_C, V_2_C, Ti_3_CN, Mo_2_C, and particularly Ti_3_C_2,_ which was the first and preferred MXene reported. It should be noted that the use of HF as etchant (either directly or formed in situ) raises important workup safety risks and toxicity concerns due to the potential for serious permanent skin burns,^[^ ^9^ ^]^ ease of HF penetration through the skin,^[^ ^9^ ^]^ and the exceptionally high toxicity of HF and fluoride in water for animals and plants.^[^ ^10^ ^]^ Furthermore, several occurrences of accidental release of HF intended for commercial use have been reported.^[^ ^11^ ^]^ These significant caustic, toxicological and environmental problems caused by HF and fluorides can be overcome by developing other fluoride‐free etchants, as is the case of the most frequently used molten salt methods. In addition, the use of solids as etchants has additional advantages over liquid phase etchants as in the case of aqueous HF solutions.

Since the outset of MXene development, their electrical properties and the activity of the early transition metals attracted considerable interest for their application in areas where the combination of these two parameters was highly desirable, particularly in electrical devices, such as batteries, supercapacitors, and electrodes in general. All this research is broadly related to renewable energies, particularly green electricity storage, an area of enormous importance. A common requirement in this context is good electrical conductivity. The electrical conductivity of MXenes can range from semiconducting to highly conductive. Recently compiled conductivity data for Ti_3_C_2_T_x_, synthesized from different fluoride‐based methods, range between 4.7 –20 000 S cm^−1^ with an average of 4 500 S cm^−1^.^[^ ^12^ ^]^ The conductivity may varying significantly depending on the synthesis conditions, the composition and surface terminations.^[^ ^2^, ^12^, ^13^, ^14^ ^]^ Regarding the importance of surface terminations on electrical conductivity, MXene samples prepared under the same conditions, a series of Nb_2_CT_x_, samples were prepared using molten salt etching and tested. It was observed that the surface termination significantly affects its conductivity, with values of 25, 60, 81, 180, 305, 345 S cm^−1^ for O, □, NH, S, Se, Cl terminal groups, respectively (where □ represents the absence/elimination of surface groups).^[^ ^13^ ^]^ Interestingly, Nb_2_CO_x_ prepared by surface substitution of Nb_2_CCl_2_,^[^ ^13^ ^]^ yielded a very similar conductivity to Nb_2_CO_0.5_(OH)_0.8_F_0.4_ (24 S cm^−1^),^[^ ^15^ ^]^ prepared by LiF  and  HCl solution etching. Electrical conductivity has brought new opportunities in the field of electrocatalysis, in which MXene‐based electrodes have become among the most promising materials for hydrogen and oxygen evolution reactions and CO_2_ and N_2_ reduction.^[^ ^16^ ^]^

In view of this considerable interest in novel MXenes and taking into account the aforementioned safety concerns and potential negative environmental impact raised by the use of concentrated HF solutions, there is an evident need to develop alternative etching procedures that should be reliable, safer, and easier to scale up to the (multi)gram scale while avoiding the use of HF. Some attempts to overcome fluoride etching include the use of corrosive hydroxides, such as NaOH and particularly tetraalkylammonium hydroxides that can simultaneously act as exfoliant agents.^[^ ^17^, ^18^, ^19^ ^]^ Other alternative etching procedures have reported the use of toxic halogens.^[^ ^20^, ^21^ ^]^ Although more amenable, electrochemical etching also suffers limitations in terms of electrode preparation, scale of the synthesis, and voltage window that can be used.^[^ ^22^, ^23^, ^24^ ^]^ Other physical etching methods include plasma treatment,^[^ ^25^ ^]^ magnetron sputtering,^[^ ^25^ ^]^ or laser ablation. We have contributed to this field by showing that the energy of laser photons delivered in the nanosecond time scale results in a sudden thermal shock on the MAX particles that also results in the generation of MXene as small sized nanoparticles.^[^ ^26^, ^27^ ^]^ The process can even be used to install arbitrary alkoxy groups as surface terminations.^[^ ^28^ ^]^ However, these all physical etching treatments have limitations in terms of adjusting operation parameters, amount of sample that can be obtained, and reliability in comparison to chemical treatments. Therefore, the previous methods suffer from limitations in their applicability and have achieved limited success. Although bottom‐up synthesis of MXenes could be an appealing solution, at the moment there was a clear gap in finding suitable etching procedures to convert MAX precursors into MXenes that has mostly solved with the use of *molten salt etching*.

Molten salt synthesis is a very well established procedure for the preparation of advanced carbon materials,^[^ ^29^ ^]^ highly crystalline perovskites, metal oxides,^[^ ^30^ ^]^ fluorides,^[^ ^31^ ^]^ and other inorganic solids,^[^ ^30^ ^]^ including metal nitrides. In the case of bulk metal nitrides, due to the difficulty to their synthesis by other methods, frequently requiring high temperatures (>1000 °C) and pressures in the GPa range, molten salt synthesis was one of the preferred procedures for the preparation of these 3D, bulk metal nitrides with large particle size and crystallinity.^[^ ^32^ ^]^ In a recent example of molten salt synthesis of 3D metal nitrides, VN, MoN, WN, and TiN were obtained at 250 °C using molten ZnCl_2_ containing Li_3_N as N source and the corresponding metal chlorides as precursors.^[^ ^33^ ^]^ A logical extension of molten salt synthesis to obtain 3D bulk nitrides is the adaptation of these methods for the preparation of MXene nitrides, particularly considering that one of the major advantages of the molten salt synthesis is its scalability.^[^ ^34^ ^]^

With these precedents on the synthesis of 3D bulk metal nitrides, the present review is exclusively focused on the molten salt etching method to prepare 2D MXenes from the corresponding MAX phase, the preparation of 3D bulk metal carbides and nitrides being excluded. As it will be shown in this review, the molten salt etching method appears to be general, reliable, providing control of the surface termination, scalable at a multigram scale, and providing directly in a single step MXene materials that are suitable for some of the most important applications of these materials, particularly as electrodes. A seminal step forward in the molten salt etching procedure was the observation of the replacement of the A element in the MAX phase using Lewis acid ZnCl_2_ molten salt and that the process can finally lead to the corresponding MXene.^[^ ^8^ ^]^ Subsequently, it was realized that the use of molten salts of transition metal halide Lewis acids is a general procedure in the synthesis of MXenes from MAX phases, valid for different A‐site metals, M elements, and transition metal halide salts (provided that there is a favorable redox couple with the A element).^[^ ^35^ ^]^ Finally, it was also shown that molten salt etching can exert control over the surface terminal groups, allowing introducing and replacing some surface groups.^[^ ^13^ ^]^ The combination of these features renders molten salt etching the most versatile and convenient MXene preparation procedure.^[^ ^36^ ^]^

We will first describe the molten salt synthesis of MXenes with examples of the versatility and advantages of this method, followed by examples of the use of molten salt procedure as post‐synthetic treatment of MXene to alter the surface functional groups. A section summarizes MXene‐derived materials prepared by molten salt with special emphasis on showing the suitability of molten salt etching for the preparation of MXene containing metal nanoparticles, metal clusters, and even MXene‐supported single atoms, a fact that appears as a more general consequence of etching with molten metal halide salts than initially expected. This is in fact how we entered in the field, by realizing the electrocatalytic efficiency of Fe single atom sites on surface modified Ti MXene to catalyze the two‐electrons reduction of O_2_ to H_2_O_2_.^[^ ^37^ ^]^ Some important applications of MXenes prepared by molten salt methods have also been briefly summarized, showing the advantages of this method for the preparation of samples suitable for their direct application in electrochemical energy storage and conversion. The last section summarizes the main points of the review and provides our views on future developments in this field, emphasizing the potential of MXenes prepared by molten salt as thermal catalysts due to the presence of supported metal/metal oxide nanoparticles and the intrinsic structural catalytic sites present in these materials.^[^ ^38^ ^]^

## 2 Molten Salts in MXene Synthesis

This section gives a historical perspective of the development of molten salt etching for MXene preparation. As commented earlier, molten salt synthesis was a well‐established synthesis procedure for 3D bulk metal nitrides and other ceramic materials. Molten cryolite at 960 °C has been known to be able to remove Si from Ti_3_SiC_2_ as early as 1999.^[^ ^39^ ^]^ A similar finding was reported in 2011 where Al could be removed from Ti_2_AlC by molten LiF at 900 °C.^[^ ^40^ ^]^ However, this resulted in cubic non‐stoichiometric TiC_x_ phases rather than the MXene in both cases. In 2016, an adaptation of this approach led to the first successful synthesis of a nitride MXene, Ti_4_N_3_T_x_, from Ti_4_AlN_3_ in molten LiF/NaF/KF at the ternary eutectic composition at 550 °C.^[^ ^41^ ^]^ The progression of the molten salt synthesis of nitride MXene is discussed in more detail in Section 2.2, while further detail on molten salt synthesis of carbide MXene is given in Section 2.1.

Following previously unrelated methods for the replacement of the A‐site of MAX phases, it was reported in 2019 that Ti_3_AlC_2_ in an excess of molten ZnCl_2_ at 550 °C transformed first into the substituted Ti_3_ZnC_2_ and then into Cl terminated Ti_3_C_2_T_x_ MXene, marking the first molten salt derived carbide MXene and the first fluorine‐free and water‐free synthesis of MXene.^[^ ^8^ ^]^ In 2020, the potential of this method was recognized and explored extensively to generalize the method with a wide array of carbide MAX phases as precursors and late‐transition metal halide salts as Lewis acidic etchants, including bromide and iodide salts, and realizing the redox‐controlled reaction of the etchant metal cation with the A‐site element as the guiding principle.^[^ ^35^ ^]^

Considering these various core contributions to the development of the use of molten salt in the synthesis of MXene from MAX phases, it is clear that it offers some major advantages over the widely used traditional fluoride‐based techniques. Some of the major differences between the HF and in situ HF techniques and molten salt etching are summarized in **Table** **1**.

**Table 1:** Comparison of MXene synthesis by MAX phase etching by molten salts to traditional fluoride‐based etching.

	MXene synthesis route		
	Concentrated HF (aq.)	In situ HF forming	Molten salt
Morphology after etching	Accordion‐like	Morphology after etching	Accordion‐like
Few‐ or single‐layer delamination	Post‐synthesis, (mainly with DMSO, TBAOH, TMAOH)	Few‐ or single‐layer delamination	Post‐synthesis, (mainly with DMSO, TBAOH, TMAOH)
Surface groups	F, O, OH; post‐etching modification with limited coverage	F, O, OH; post‐etching modification with limited coverage	Depending on the Lewis acid salt etchant. Most commonly Cl. Also, F, Br
Reagent toxicity	Very high safety risk (skin ulcers and through skin) Environmental toxicity of high F concentration in water for animals and plants	Less severe skin burns Environmental toxicity of high F concentration in water for animals and plants	Possible to use of non‐fluorine chemicals Use of solid chemicals Lower environmental toxicity
Hybridization (nanoparticles, single‐atoms)	Post‐synthesis	Post‐synthesis	One‐step hybridization with metal nanoparticles. Easy single‐atom installment
Limitations on scalability	HF safety and corrosion concerns, Limited yield from large liquid volumes	HF safety and corrosion concerns, Limited yield from large liquid volumes	Easy scale‐up at multigram amounts

### 2.1 Molten Salt Derived Carbide MXene

Although post‐synthetic modification of the MAX phase to obtain other different MA'X phases was initially not a general methodology, it may work in the case of certain MAX phases using molten salts. In this regard, Huang and coworkers reported that Lewis acid ZnCl_2_ molten salt (m.p. 280 °C) is able to convert, among others, Ti_3_AlC_2_ and Ti_2_AlC into Ti_3_ZnC_2_ and Ti_2_ZnC.^[^ ^8^ ^]^ An important point to remark is the absence of any fluorine‐containing compound in the process, therefore, greatly mitigating the environmental concerns caused by the use of F containing chemicals. It is worth commenting that these Zn MAX phases, as well as other MAX phases in which A is a late transition metal, cannot be prepared directly by metallurgic solid‐state synthesis at high temperatures over 1500 °C since they are not thermodynamically stable in comparison to metal alloys and other phases, or the metal can evaporate at these high temperatures. However, the exchange of the Al MAX phase can occur at much lower temperatures (550 °C) than those required for the direct synthesis, thus allowing their formation. The process is, however, not general, and it fails to obtain the Zn MAX phases of Al MAX nitrides. This is probably a consequence of the stronger M─A bonding in nitrides as compared to carbides. It would be important if an analogous post‐synthetic MAX to MA'X exchange process could be equally developed for nitrides. The A‐site exchange process also fails in the case of V_2_AlC. In fact, there are still no precedents on the use of the molten salt method to the synthesis of V_2_C, this being a gap to be filled in this area. If the proportion of ZnCl_2_‐to‐Al MAX is increased from 1.5 to 6, then the transformation of Ti_3_AlC_2_ and Ti_2_AlC does not stop at the Zn MAX phase. Instead, after the Al/Zn exchange, the reaction continues to form the corresponding MXenes with Cl‐terminal groups and Zn metal intercalation.^[^ ^8^ ^]^ Herewith it was recognized for the first time that a water‐free and fluorine‐free molten salt with strong Lewis acidity could selectively etch Al from Ti_3_AlC_2_ to produce Ti_3_C_2_T_z_ with a content of Cl as surface termination near a theoretical maximum corresponding to Ti_3_C_2_Cl_2_. The phase evolution of MAX to MXene was followed over different reaction times by XRD, and HAADF with EDS analysis, showing that the Cl termination occurs as a core‐shell reaction. Similar conclusions have been reached by *operando* synchrotron radiation XRD.^[^ ^42^ ^]^ Equations ((1), (2), (3)) summarize the process. Easy removal of the Zn by aqueous HCl renders Ti_3_C_2_Cl_2_ and Ti_2_CCl_2._

(1) $$Ti_{3}AlC_{2}+1.5\;ZnCl_{2}\to Ti_{3}ZnC_{2}+0.5\;Zn+AlCl_{3}$$

(2) $$Ti_{3}AlC_{2}+1.5\;ZnCl_{2}\to Ti_{3}C_{2}+1.5\;Zn+AlCl_{3}$$

(3) $$Ti_{3}C_{2}+Zn\to Ti_{3}ZnC_{2}$$

**Figure 2:**
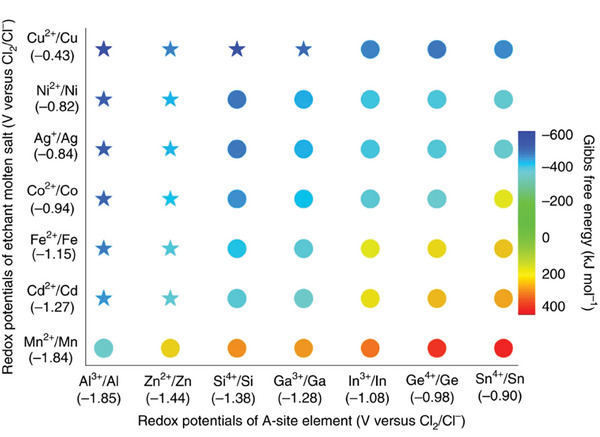
Gibbs free energy chart predicting (blue color) those MAX phases whose A element can be etched by metal chlorides to form the corresponding MXene due to the occurrence of chemical oxidation. The stars indicate those cases in which the concept of redox‐active metal cation Lewis acid chloride has been proved experimentally. Adapted with permission.^[^ ^35^ ^]^ Copyright 2020, Springer Nature.

It happens, however, that ZnCl_2_ only converts a few MAX phases into MXenes. As a way to expand the applicability of Lewis acid molten salts, it has been proposed that the etching of the A element is favored if the cation of the Lewis acid molten salt is able to promote the chemical oxidation of the A element according to Equation (4).^[^ ^35^ ^]^ Hence, the successful synthesis of MXenes from various MAX phases with different A‐site elements using a wide range of molten salt Lewis acid chloride etchants could be predicted based on the redox potential of the active cation in the molten salt relative to that of the A‐site element (**Figure** **2**). In this way, the A element of the MAX phase should become oxidized, and the Lewis metal cation reduced to the metal state. Several of these predicted combinations for MAX phase and Lewis acid chloride molten salt have been confirmed experimentally. Furthermore, the procedure was extended to include other halide salts, including examples using CuI and CuBr_2_,^[^ ^35^ ^]^ or MAX phases with 1D or 2D morphology.^[^ ^43^ ^]^ These findings have thus cemented the use of Lewis acid molten halide salts as MAX phase etchants as a general and flexible procedure for the synthesis of MXene.

(4) $$A+\frac{x}{y}\;BCl_{y}\to ACl_{x}+\frac{x}{y}B$$

According to Equation (4), etching with a redox‐active Lewis acid chloride generates another metal that remains in the material. Thus, for instance, in the case of using CuCl_2_ to etch Ti_3_SiC_2_, Cu metal spheres appear on the accordion‐like MXene material. These Cu spheres can be removed from the MXene phase by oxidative dissolution with ammonium persulfate (APS). However, in addition to Cu removal, this oxidative treatment with APS introduces O as the predominant surface termination group, with Cl becoming a close secondary surface termination group, as evidenced by EDS, TPD‐MS, and XPS analysis.^[^ ^35^ ^]^ Over time (c.a. 12 h), such oxidative treatments can even cause damage to the MXene structure through gradual phase evolution from the MXene crystal structure to an amorphous M oxide.^[^ ^42^ ^]^

As previously noted, molten salts of anions other than Cl^−^, and particularly Br^−^ and I^−^, can work similarly, resulting in a different surface functionalization.^[^ ^35^, ^44^ ^]^ Thus far, CuBr_2,_ and CuI have been the preferred molten salts for etching the A element of a MAX phase to convert it into the Br and I terminated MXenes.^[^ ^35^, ^44^ ^]^ The process is summarized in Equations (5) and (6). Using Cu halides, the Cu metal formed in the process can be removed from the MXene solid using either various oxidizing reagents or NH_4_Cl/NH_3_ aqueous solution in the presence of oxygen. The latter treatment maintains a high degree of halide functionalization.^[^ ^44^ ^]^

(5) $$Ti_{3}AlC_{2}+2.5\;CuX_{2}\to Ti_{3}C_{2}X_{2}+2.5\;Cu+AlX_{3}$$

(6) $$Ti_{3}AlC_{2}+5\;CuI\to Ti_{3}C_{2}I_{2}+5\;Cu+AlI_{3}$$

Regarding the mechanism through which CuCl_2_ etching in molten alkali chloride operates, a detailed study of Nb_2_GaC etching by *operando* synchrotron radiation XRD has revealed that the process takes place through a series of temperature/time dependent steps.^[^ ^42^ ^]^ The first process consists in the expansion of Nb_2_GaC crystal, followed by an easy, complete and super‐fast replacement of Ga by Cu, forming Nb_2_CuC in the temperature range from 460–480 °C.^[^ ^42^ ^]^ Higher temperatures further expand the Nb_2_CuC lattice and, at ≈600 °C, Nb_2_CuC is gradually converted into Nb_2_CCl_2_, the Cl termination progressing from the periphery to the center of the particles wherein Nb_2_CuC can still be present. The XRD peaks corresponding to Nb_2_CCl_2_ reached a maximum in intensity when Nb_2_CuC has disappeared and extending the time at 750 °C decreases the crystallinity of the Nb_2_CCl_2_ sample.^[^ ^42^ ^]^ As commented earlier, in the case of ZnCl_2_ and the observation of the formation of Ti_3_ZnC_2_ and Ti_2_ZnC MAX phases from Ti_3_AlC_2_ and Ti_2_AlC phases, this *operando* XRD study indicates that this MAX phase transformation prior to etching can be very general and can even serve to prepare new MAX phases by solid‐state reaction. This has recently been confirmed by proposing a “*chemical scissor*” method based on Lewis acid molten salt to convert a MAX phase into other different MAX phases, replacing the A‐site composition.^[^ ^36^ ^]^ It appears now evident that optimal conditions of molten salt etching regarding temperature and time should be determined in each case to achieve the most crystalline material as, for instance, in the case of Nb_2_GaC etching to Nb_2_CCl_2_ 750 °C for 30 min.^[^ ^42^ ^]^

One of the advantages of the reported molten salt etching is the possibility of installing the corresponding halide as surface termination groups as well as binary or ternary mixtures of them, such as Cl/Br, Br/I, or Cl/Br/I, in variable proportions.^[^ ^44^ ^]^ In the case of a single halide, after the removal of Cu, EDS analysis determined a 3:2 stoichiometry for Ti and halide, supporting the ideal, theoretical Ti_3_C_2_X_2_ (X: Cl, Br or I) composition for the resulting MXene. The nature of the halide influences the distance among the individual MXene sheets in the accordion‐like loose stack, as determined by XRD, with c lattice parameters of 22.22 to 25.00 Å for Cl and I, respectively.^[^ ^44^ ^]^ This interlayer distance is larger as the atomic size of the halide increases, and it has intermediate values between the two unitary surface terminal groups for the case of MXenes with binary halides. The CuBr_2_ and CuI molten salt etched Ti_3_C_2_ exhibits improved performance as cathodes in Zn‐ion batteries compared to Cl‐terminated and the traditional HF‐etched MXene.^[^ ^44^ ^]^

Most frequently, the molten salt etching method results in an accordion‐like MXene phase that, even though the A element has been etched, cannot be easily exfoliated to the single sheet material due to the strong van der Waals interaction among the sheets. The occurrence of a complete transformation of the MAX phase into MXene can be determined by chemical analysis of the A element and by the disappearance of the XRD peaks corresponding to the MAX crystals. Consequently, these stacked MXene clays do not generally disperse well in aqueous medium. MXene dispersion in water at the single or few‐layer exfoliation level is highly desirable in many important applications related to the use of MXene as electrodes, supercapacitors, and others.

**Figure 3:**
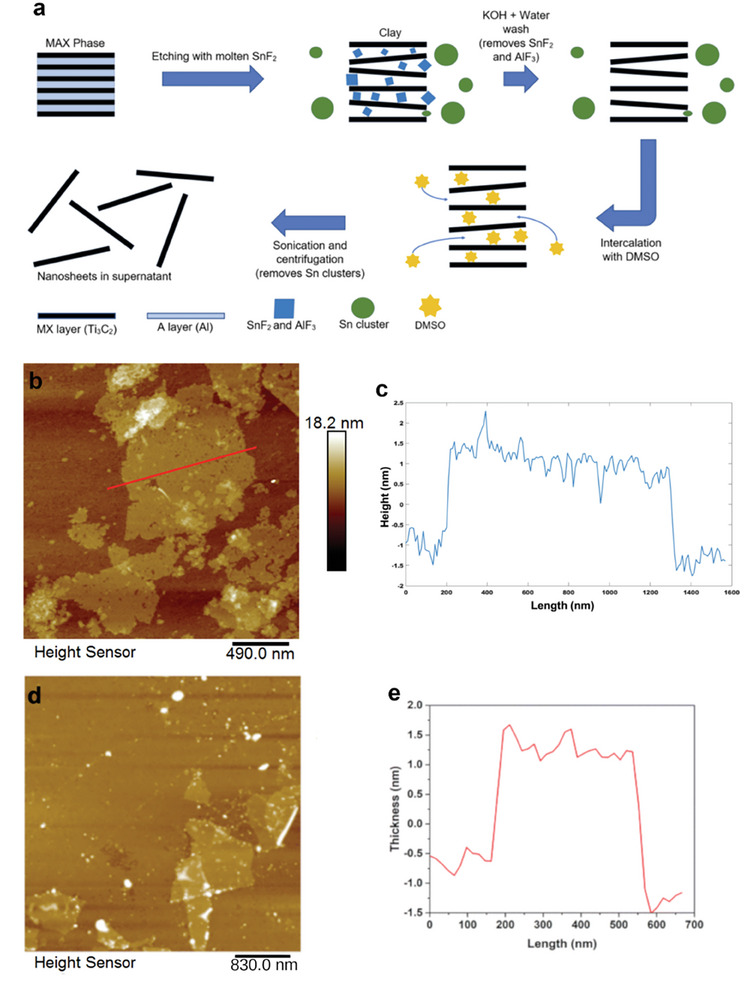
a) Steps for the SnF_2_ etching of Ti_3_AlC_2_ and dispersion of the resulting Ti_3_C_2_T_x_ MXene clay. b) Frontal AFM image of a sheet from an aqueous Ti_3_C_2_T_x_ suspension prepared by the molten salt SnF_2_ etching of Ti_3_AlC_2_ exfoliation. c) Vertical profile along the red line marked in the frontal AFM image of Ti_3_C_2_T_x_. Adapted with permission.^[^ ^45^ ^]^ Copyright 2021, Elsevier. d) AFM image of Nb_2_CT_x_ obtained by SnF_2_ etching and KOH treatment. e) Vertical profile showing the thickness of one Nb_2_CT_x_ sheet. Adapted with permission.^[^ ^46^ ^]^ Copyright 2022, Royal Society of Chemistry.

To overcome this limitation, Green and coworkers reported for Ti_3_AlC_2_ the use of SnF_2_ molten salt (m.p. 213 °C) in six‐fold molar excess at 550 °C as etchant reagent, resulting in MXene containing Sn metal.^[^ ^45^ ^]^ Subsequent removal of Sn metal and the excess of SnF_2_ salt by 0.1 m aqueous KOH leads to a MXene stack. This was further soaked in dimethyl sulfoxide (DMSO) for 20 h and washed several times with water before ultrasound treatment in water. The final MXene yield is ≈10%. The process is illustrated in **Figure** **3**a. Based on EDS analysis and the detection of Sn between the MXene sheets, it is claimed that SnF_2_ intercalates within the MXene sheets, and the large Sn atomic radius results in an expansion of the inter‐layer distance.^[^ ^45^ ^]^ Importantly, water compatibility arises from the partial replacement of F surface terminal groups by O and OH in the KOH treatment step to remove Sn.^[^ ^45^ ^]^ The resulting Ti_3_C_2_(F, O, OH) suspension is indefinitely persistent in H_2_O, likely to be largely mono‐ or few‐layers, with an average lateral size dimension of 359 nm and a negative particle charge with a zeta potential of −31.7 V. Figure 3b shows an AFM measurement of sheet thickness that corresponds to a Ti_3_C_2_ monolayer.^[^ ^45^ ^]^

A similar two‐step SnF_2_ molten salt etching at 750 °C and subsequent KOH treatment has been reported for the synthesis of Nb_2_CT_z_ (10–15% yield).^[^ ^46^ ^]^ Using isopropylamine as the intercalating agent in water, nanosheets of 1.5 nm thick, 270 nm lateral size, and – 41.3 V of zeta potential were obtained (Figure 3d,e).^[^ ^46^ ^]^ One interesting property of the SnF_2_ molten salt‐prepared Nb_2_C samples is their higher stability against spontaneous oxidation compared to analogous samples prepared by HF etching.^[^ ^46^ ^]^ Following the absorption intensity at 776 nm of Nb_2_C over time, it was found that aqueous suspensions of Nb_2_C samples prepared by SnF_2_ etching undergo a 23% decrease in absorbance in 200 h, while analogous dispersions of the Nb_2_C prepared by HF etching are converted to Nb oxides, with a 58% decrease in absorbance for the same time.^[^ ^46^ ^]^ Considering the reported tendency of Nb_2_C to undergo oxidation to Nb oxides,^[^ ^47^ ^]^ this influence of the surface terminal groups is important and points to a higher degree of stability against oxidation which could be achieved in Nb_2_C MXenes by having appropriate surface functional groups. Further understanding of how certain surface groups stabilize Nb_2_C against oxidation is, however, still needed.

Besides SnF_2_, SnCl_2_ is also a suitable Lewis acid to promote Al etching, with a mixture of NaCl and KCl molten salt employed as flux.^[^ ^48^ ^]^ Using this procedure to etch Ti_3_AlC_2_, it was found that the residual small Sn metal nanoparticles present in the resulting Ti_3_C_2_Cl_2_ MXene are useful to avoid the tight stacking of the accordion‐like MXene phase, thereby allowing the intercalation of Li^+^.^[^ ^48^ ^]^ As a consequence of the presence of small Sn nanoparticles in the Ti_3_C_2_Cl_2_ material, the interlayer distance was found to expand from the expected 9.29 to 10.8 Å for the Sn/Ti_3_C_2_Cl_2_ solid.^[^ ^48^ ^]^ In this way, the resulting Sn/Ti_3_C_2_Cl_2_ can be used directly as an anode in Li‐ion batteries. Obviously, the SnCl_2_‐assisted, one‐step process is much more convenient than the preparation of Ti_3_C_2_ by conventional HF etching and subsequent addition of Sn metal precursor by wet chemical routes. Another advantage of the molten salt‐assisted method is the minimization of Ti_3_C_2_Cl_2_ oxidation.^[^ ^48^ ^]^

**Figure 4:**
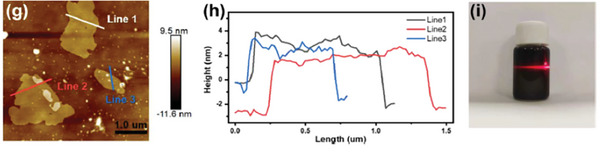
AFM image (left) and representative sheet thickness measurements (center) of Ti_3_C_2_Cl_2_ obtained by SnCl_2_ etching in NaCl‐KCl molten salt along three lines indicated by the color. The right panel shows the Tyndall effect of a red laser beam through a dispersion of exfoliated Ti_3_C_2_Cl_2_. Adapted with permission.^[^ ^48^ ^]^ Copyright 2022, Elsevier.

TEM confirmed the small size of Sn nanoparticles and their presence at the borders and within the Ti_3_C_2_Cl_2_ sheets.^[^ ^48^ ^]^ A study of the influence of the temperature on the SnCl_2_ etching process, monitored by XRD following the (002) peak of MAX/MXene phases and the characteristic Sn diffractions, shows that the Ti_3_C_2_Cl_2_ MXene develops gradually with the temperature and the MXene sheets control Sn particle growth up to 600 °C, beyond which obvious Sn agglomeration takes place.^[^ ^48^ ^]^ Temperatures above the SnCl_2_ boiling point (>700 °C) diminish SnCl_2_ etching, probably due to the evaporation of this etching reagent. AFM measurements after ultrasound treatment of the etched Ti_3_C_2_Cl_2_ sample show that the sheet thickness is between 2 and 4 nm, thus corresponding to a few sheets stacking.^[^ ^48^ ^]^ Water suspension of Ti_3_C_2_Cl_2_ was persistent, exhibiting the Tyndall effect, thus, indicating high hydrophilicity of the sample. **Figure** **4** shows an AFM image of Ti_3_C_2_Cl_2_ etched by SnCl_2_/(NaCl‐KCl) with the thickness measurement of some nanoplatelets and a photograph of the aqueous dispersion.

By a modification of the original ZnCl_2_ molten salt etching procedure, it has been possible to prepare, a series of MXenes, including Ti_3_C_2_T_x_, Nb_2_CT_x_, Nb_4_C_3_T_x_, Ta_2_CT_x_, Ti_2_NT_x_, Ti_3_CNT_x_, directly from the MAX phase.^[^ ^49^ ^]^ The process combines Al by Zn exchange and subsequent AlCl_3_ and Zn metal evaporation in a single thermal treatment. The reaction is performed under vacuum in a furnace in the range of temperatures from 550 to 700 °C. Evaporation of resulting AlCl_3_ and Zn can be completed at the optimal temperature leaving MXene as residue. The resulting MXene obtained under the best conditions exhibits a high specific surface area and undergoes easy exfoliation due to the increased hydrophilicity of the material. Overshooting the optimal temperature (700 °C in the case of Ti_3_C_2_) is detrimental due to damage to the MXene sheets.

**Figure 5:**
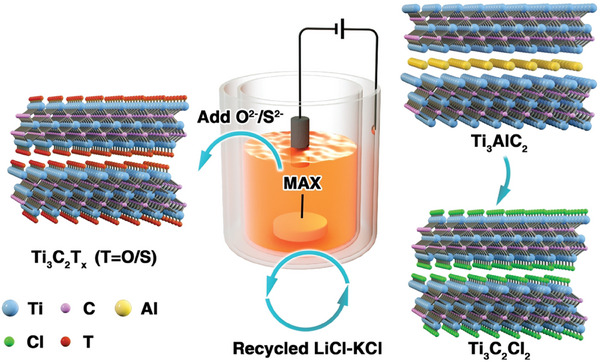
Illustration of the molten salt‐assisted electrochemical etching allowing the control of the surface termination. Reproduced with permission.^[^ ^50^ ^]^ Copyright 2021, Wiley‐VCH.

An analogous concept of assistance of the etching process by oxidation of the A atom of the MAX phase, but realized in a different way, is the molten salt‐assisted electrochemical etching.^[^ ^50^ ^]^ In this method, the MAX phase is used as the anode and nickel as the cathode of an electrochemical cell having molten LiCl‐KCl flux at 450 °C as electrolyte. The process is illustrated in **Figure** **5**. By applying 2.0 V to the cell and using Ti_3_AlC_2_ as the MAX phase in the presence of Cl^−^, Al is converted into AlCl_3_, being the easiest element to oxidize, and under the conditions of the electrochemical reaction and high temperature, it evaporates from the flux at the operating temperature resulting in a Cl‐terminated Ti_3_C_2_Cl_2_ MXene. Voltages higher than 3 V also result in the attack of Ti besides Al and the unwanted formation of TiCl_4_, while lower voltages, such as 1.6 V, leave most of the Ti_3_AlC_2_ unaltered. At the appropriate operating voltage in the 1.6–3.0 voltage range, a complete Al etching with the formation of the Cl‐terminated MXene can be obtained. It is claimed that this electrochemically assisted, rather than chemical oxidation, is more convenient from the sustainability point of view since it does not require chemicals, and it does not generate large volumes of corrosive wastes formed during the removal of metals from the MXene material.^[^ ^50^ ^]^ However, a lifecycle assessment, considering the electricity generation and other components of the electrodes, would also have to be considered.

The electrochemically‐assisted molten salt etching method has also proven to be successful for Ti_3_SiC_2_ etching.^[^ ^50^ ^]^ It also allows the selection of the surface termination group by introducing, once the electrochemical etching is complete, a pressed pellet of a Li compound in the hot melt.^[^ ^50^ ^]^ The possibility of the controlled surface termination was proven with Li_2_O and Li_2_S, although in the last case, O in an undetermined proportion was accompanied by the intended S as the termination group.

The quality of the MXene samples obtained by this alkali metal halide mixture molten salt and the wide range of groups that can be introduced have allowed a detailed study of the properties of the resulting MXenes as a function of the surface termination. This study has confirmed the remarkable influence on the MXene properties of the surface termination groups that cannot be considered to be inert components of the MXene, simply fulfilling the bond requirements. Particularly it was determined that the structural stress and deformations derived from the bulkiness of the surface terminal groups are more notable in the case of MXenes with fewer layers, such as Ti_2_C. This stress can result in changes in the relative position on the surface of termination groups. Properties such as electrical conductivity, the appearance of superconducting behavior, and magnetic susceptibility are all deeply influenced by these surface termination groups.^[^ ^13^ ^]^

MXenes offer a large range of possibilities in compositional combinations regarding the early transition metal, the X layer, and surface termination groups. The use of molten salt to prepare multi‐metallic MXenes represents one of these possibilities that have yet to be fully exploited and appears to be promising to further modulate and control the electronic properties of MXenes. CuCl_2_ molten salt etching has recently been used to obtain medium/high entropy MXene alloys from medium/high entropy Ga MAX phases.^[^ ^51^ ^]^ Further synthesis of these high entropy alloys MXenes can eventually lead to much enhanced and optimal performance of MXenes for most applications of these materials.

Some MXenes are prone to oxidation under the harsh conditions employed during etching. A molten salt‐shielded synthesis under air has been reported, based on first preparing a pellet of Ti_3_AlC_2_ and NaCl/KCl mixture and, then, covering the pellet in a crucible with additional NaCl/KCl containing CuCl_2_ as etching agent in the proportions corresponding to the eutectic composition.^[^ ^52^ ^]^ The etching is carried out under air at 700 °C, with the molten salt providing a shield to avoid oxygen attack to the MXene sheets being formed at this high temperature. After etching (10–40 min), the Cu metal formed on the MXene can be removed by any of the procedures commented on earlier for other Cu metal‐containing MXenes prepared using CuCl_2_ molten salt, for example, by oxidation with a solution of APS. Besides Ti_3_C_2_, the molten salt shielding effect was also used to prepare Ti_4_N_3_, Ti_3_CN, and Ti_2_C MXenes in 70, 78, and 65% yield, respectively.^[^ ^52^ ^]^ Note that, except Ti_3_CN, nitride MXenes are typically difficult to obtain using most of the reported wet chemical MXene synthesis methods. The process was scaled up for a large 60 g batch, resulting in samples with identical MXene characterization data as those of 0.5 g batch samples. For a 20 g batch of Ti_3_AlC_2,_ a 72 % yield of the Ti_3_C_2_ MXene was reported.^[^ ^52^ ^]^ Besides preparation procedures and external conditions, a recent study has shown that internal stability factors, particularly the M─T bond energy, strongly influence MXene stability, with multilayer Ti_3_C_2_Br_z_ becoming oxidized faster than Ti_3_C_2_Cl_z_.^[^ ^53^ ^]^ The degradation study of Ti_3_C_2_T_z_ was carried out in water suspension at high pH and high temperature over several days, monitoring the growth of the characteristic TiO_2_ absorption band in the UV–vis spectra of the suspensions.

### 2.2 Molten Salt Derived Nitride MXene

**Figure 6:**
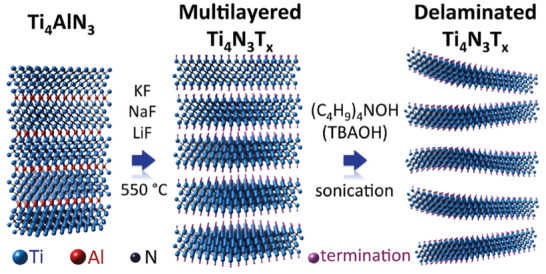
Molten salt assisted method to etch Ti_4_AlN_3_ using a eutectic mixture of alkali fluorides. Subsequent sonication in the presence of tetrabutylammonium hydroxide renders exfoliated Ti_4_N_3_ MXene. Reproduced with permission.^[^ ^41^ ^]^ Copyright 2016, The Royal Society of Chemistry.

MXene nitrides are more difficult to prepare than the corresponding MXene carbides, the main reason being the lower stability of the M─N bonds and the stronger M‐A interactions in nitrides. This lower stability of MXene nitrides causes their dissolution under the typical HF etching used for the corresponding carbides.^[^ ^54^ ^]^ For this reason and considering that the electronic conductivity of nitrides are generally higher than their corresponding carbides,^[^ ^55^ ^]^ there is considerable interest in developing general etching procedures and adequate exfoliation methods for nitrides. To overcome this limitation, Gogotsi and coworkers reported, for the first time, that molten salts of alkali metal fluorides in eutectic proportions (LiF, NaF, and KF in 29, 12, and 59 wt.%) at 550 °C are able to selectively etch Al in Ti_4_AlN_3_, resulting in the corresponding Ti_4_N_3_T_z_ MXene.^[^ ^41^ ^]^ This discovery marked a major divergence from the ubiquitous HF or in situ HF forming etching methods. Although fluoride salts are still used, the workup with solids still represents a considerable safety advantage when compared to the use of fluoride in aqueous solutions. Subsequent sonication of the Ti_4_N_3_ MXene intercalated with tetrabutylammonium hydroxide (TBAOH) renders the delaminated material. The process is illustrated in **Figure** **6**. The selective Al etching was confirmed by XRD based on the observation of two peaks at 2θ 6.3 and 12.7 ° corresponding to Ti_4_N_3_. Although Al was detected by EDS analysis of SEM images, it was attributed to the presence of Al fluorides. The formation of Ti_4_N_3_ was confirmed by TEM observing the single crystal structure with a hexagonal arrangement of the Ti_4_N_3_ nitride flakes, with an a‐lattice parameter of 2.9 Å. It can be considered that the use of LiF, NaF, and KF in eutectic proportions for Ti_4_AlN_3_ etching is the first precedent in molten salt etching, although the use of fluorides remains a disadvantage to be overcome.

Djire et al. observed that Ti_2_NT_x_ could be synthesized from Ti_2_AlN in an “oxygen‐assisted” molten fluoride salt treatment with LiF/NaF/KF at 550 °C under gas flux of 1% O_2_ in N_2._^[56]^ This adaptation came after no MXene product could be obtained from Ti_2_AlN for the same treatment under inert gas flux (N_2_ or Ar). Analysis of the XRD spectrum of the O_2_‐asisted molten salt treated sample identified various aluminum fluoride compounds along with a shift of the (002) reflection, indicating successful removal of Al from the MAX phase A‐site and formation of the Ti_2_NT_x_ MXene. After removal of the aluminum fluoride compounds by treatment in acid, analysis of the sample by STEM with EDX confirms the absence of Al, and the presence of Ti, N, and O, consistent with Ti_2_NT_x_ with O/OH surface groups.

As previously noted, Ti_4_N_3_T_x_ MXene could be synthesized in a molten salt shielded synthesis by etching the Ti_4_AlN_3_ MAX phase in CuCl_2_ in the NaCl/KCl eutectic mixture at 700 °C in air.^[^ ^52^ ^]^ We find no prior reports on the synthesis of nitride MXene using fluoride‐free molten salts. Considering the considerable XPS intensity at the Ti 2p and O 1s binding energies associated to Ti─O, and the limited Cl detection by EDS, there is reason to believe that oxygen plays an important role in nitride MXene synthesis in molten salt.

**Figure 7:**
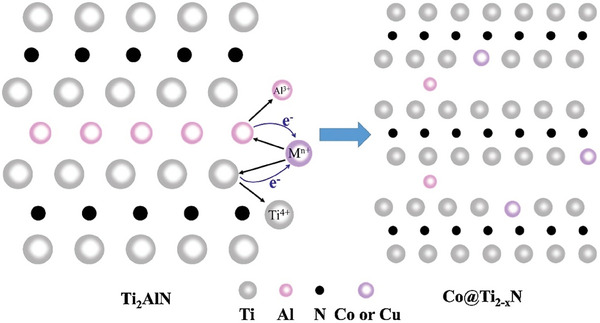
Proposed mechanism and structure of Co and Cu single atoms on Ti_2_N MXene. Reproduced with permission.^[^ ^60^ ^]^ Copyright 2021, Elsevier.

One important feature deserving additional research effort is the generation of M defects during etching of the MAX phase. Defects are well known to be responsible for the catalytic activity of many materials, and many other properties, such as conductivity, are influenced by the presence of defects.^[^ ^12^, ^57^ ^]^ The generation of defects has been reported to occur during HCl/LiF etching.^[^ ^58^, ^59^ ^]^ In one of the possibilities offered by the presence of M vacancies, it has been claimed that Co and Cu single atoms can be installed on Ti_2_N during the molten salt etching of the Ti_2_AlN phase.^[^ ^60^ ^]^ Using CoCl_2_ or CuCl_2_ as Lewis acid molten salts for Al etching and after removal of the excess of Co or Cu with HCl or HCl/FeCl_3_, respectively, the resulting samples present a residual 1.3 or 1.8 at% of Co or Cu, respectively.^[^ ^60^ ^]^ Deep characterization by aberration‐corrected high‐angle annular dark field STEM revealed the presence of Co or Cu as single atoms, occupying the lattice positions left by Ti vacancies (**Figure** **7**). The presence of single atoms was also supported by XANES measurements that indicate that the transition metal oxidation state is between 0 and +2. These single Co atoms installed on Ti_2_N are very efficient catalysts for peroxymonosulfate decomposition into hydroxyl and sulfate radicals. It is worth noting that it has subsequently been reported that Cu single‐atoms could also be anchored on Ti_3_C_2_T_x_ by etching Ti_3_AlC_2_ with molten CuCl_2_.^[61]^ This is discussed in more detail in Section 6.

## 3 Post‐Synthesis Processing

### 3.1 Metal Removal

Molten salt etching generates in the process metal residues on the MXene clay. Reduced metal particles deposited during molten salt etching usually need to be removed to obtain the target MXene for subsequent application. This necessary metal removal is one of the steps in the molten salt synthesis of MXenes that can make the whole process less convenient than desirable. The methods and reagents that can successfully purify the MXene from the product mixture resulting from the molten salt etching invariably depend on the metal species present and must be compatible with the preservation of the MXene structure. Various aqueous solutions have been employed to leach metal particles selectively.

In one of the first examples of MAX phase etching using a molten late transition metal chloride salt, bulk Zn metal was deposited from the ZnCl_2_ etchant and could subsequently be removed using 5 wt.% HCl.^[^ ^8^ ^]^ However, residual Zn could be detected by ICP‐OES and EDS of the solid after HCl washing. Based on XRD data of the as formed and purified MXene product, the presence of Zn was attributed to the incomplete conversion of the intermediate Ti_3_ZnC_2_ MAX phase to the Ti_3_C_2_Cl_2_ MXene, rather than to the presence of residual Zn metal nanoparticles.

Simple acid washings with HCl have been shown to be effective in removing bulk particles of metallic Cd (from Ti_2_CT_x_, Ti_3_C_2_T_x_, and Nb_2_CT_x_),^[^ ^13^ ^]^ and Co (from Ti_2_NT_x_),^[^ ^60^ ^]^ deposited from CdCl_2_ and CoCl_2_ etchants, respectively. Furthermore, to avoid surface group exchange, concentrated HBr was used to remove metallic Cd when CdBr_2_ was used as the etching agent.^[^ ^13^ ^]^

**Figure 8:**
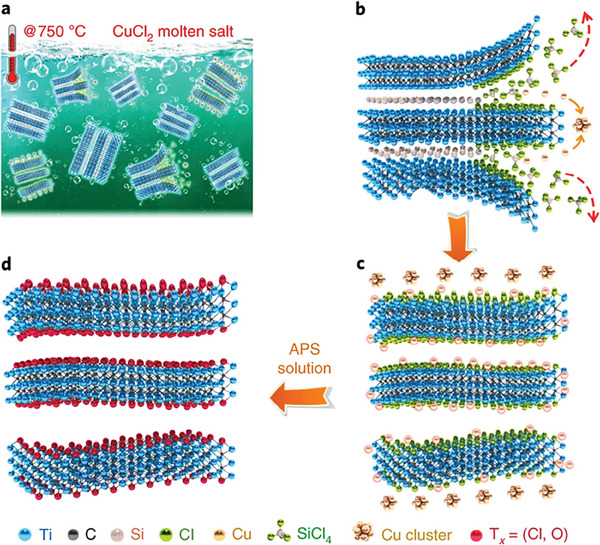
Illustration of the process of Lewis‐acid molten salt etching of Ti_3_SiC_2_ by CuCl_2_ showing the redox reaction of Cu^2+^ with Al (b), resulting in the formation of SiCl_4_(g) and metallic Cu (c). The MXene clay is subsequently treated with APS to dissolve Cu NPs. Reproduced with permission.^[^ ^35^ ^]^ Copyright 2020, Springer Nature.

With the expanded use of a greater variety of molten salts of more late transition metal halides or main group metal halides used for MAX phase etching to MXene,^[^ ^35^ ^]^ effective removal of more redox‐stable metal species was needed. Therewith, an example was given for the removal of metallic Cu after etching of Ti_3_SiC_2_ with CuCl_2_, using an aqueous solution of 0.1 m APS, selected for its high redox potential (E^0^ = +2.0 V vs NHE) (**Figure** **8**).^[^ ^35^ ^]^ However, this treatment appears to effectively remove the bulk of metal particles left after MXene synthesis using other late transition metal salts, including Cd, Fe, Ni, Co, and even Ag, which has comparatively high oxidation stability. As a side effect of the APS treatment, a high degree of substitution of surface Cl groups by oxygen also occurs.^[^ ^35^, ^62^, ^63^ ^]^ The composition for the molten salt MXene after persulfate oxidation was estimated to be Ti_3_C_2_Cl_0.77_O_1.71_.^[^ ^35^ ^]^ It has to be commented at this point that APS treatment may not be as selective in its function as expected, being able to alter the MXene structure. A detailed XRD, TEM, and XPS analysis has shown that in the case of Nb_2_CCl_2_, beyond, and optimal time, APS can damage MXene crystallinity.^[^ ^42^ ^]^

Prolonged APS treatment converts highly crystalline Nb_2_CCl_2_ samples into a disordered structure with Nb atoms in the +V oxidation state.^[^ ^42^ ^]^ As an alternative to APS to remove Cu, FeCl_3_, and NH_4_Cl/NH_3_·H_2_O (in the presence of oxygen) were also tested.^[^ ^35^, ^44^, ^60^ ^]^ Compared to APS, O_2_ in buffered ammonia is a milder oxidation agent that should be compatible with a wider range of MXenes and surface termination groups.

Zifeng and co‐workers compared different post‐synthesis treatments for the removal of the metallic Ni particles formed during the synthesis of Ti_3_C_2_Cl_x_, including aqueous solutions of H_2_SO_4_, NaOH, or APS, as well as magnetic separation.^[^ ^63^ ^]^ Small amounts of Ni were detected in both XRD and XPS measurements after treatment in NaOH solution. Furthermore, it is noted that Ni hydroxide species may also form from the reaction of residual NiCl_2_ and NaOH. For the sample treated with NaOH, an increased XPS signal (compared to the other treatments) corresponding to Ti‐O was attributed to the degradation of the MXene to TiO_2_. All treatment methods could maintain a high degree of surface Cl functionalization, with Ti to Cl atomic ratios in the range of 3 to ≈1.6–1.8. The worst case appeared to be the APS treatment for which XPS revealed a comparatively larger O 1s peak area due to the increase of the C‐Ti‐O_x_ component, indicating an increased O functionalization. Interestingly, while the dissolution of Ni by the various aqueous reagents could remove the excess Ni, magnetic separation was just as effective in separating Ni particles while preserving sample crystallinity, which can be deteriorated in the chemical treatments.

**Figure 9:**
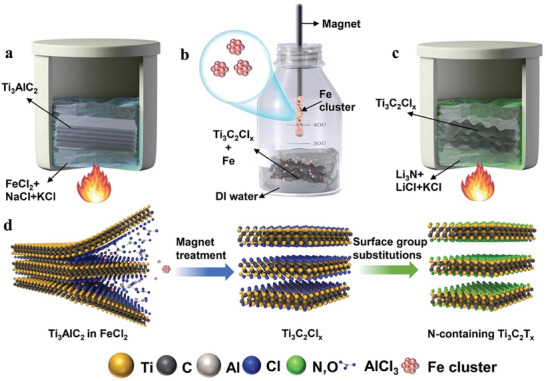
Illustration of the synthesis procedure of N‐containing Ti_3_C_2_T_x_ via molten salt etching of Ti_3_AlC_2_ MAX phase with FeCl_2_ in NaCl/KCl eutectic, followed by magnetic separation of reduced Fe particles, and treatment with Li_3_N in LiCl/KCl eutectic. Reproduced with permission.^[^ ^62^ ^]^ Copyright 2022, Wiley‐VCH.

Successful magnetic separation and APS treatment are also efficient for Fe removal from Ti_3_C_2_Cl_x_ (**Figure** **9**).^[^ ^62^ ^]^ Comparison of the two treatments showed that Fe could be reduced to trace amounts by both methods. However, a high Cl content, near the stoichiometric Ti:Cl ratio of 3:2 was only maintained by the magnetic separation of Fe from the MXene, while APS treatment produced partial O substitution, as supported by EDS, FTIR, and EELS data. Furthermore, the magnet‐treated sample showed higher thermal stability compared to the APS‐treated sample.

From the existing reports, it is clear that the removal of the metal generated in the molten salt etching from MXene may alter the surface termination. Further clarification on the effects of reagent choice and its concentration on the resulting MXene surface composition is needed, especially considering the wide range of surface groups and their different reactivity for substitution depending on the post‐synthetic treatment.

### 3.2 Delamination of Multilayered MXene Structure

Etching of MAX phases produces the removal of the A element and substitution of the M─A bonds by surface terminations, resulting in multi‐layered carbide/nitride (M_n_X_n‐1_) MXene structures with weakened out‐of‐plane interactions, often resulting in the accordion‐like particle morphology.^[^ ^1^ ^]^ Thus, even after complete A removal, MXenes still largely retain their multilayered structures (space group P63/mmc and P‐3m1) inherited from their MAX phase precursors through interplanar van der Waals bonds and, in some cases, hydrogen bonds between MXene layers.^[^ ^13^, ^64^ ^]^ The often‐seen accordion‐like morphology results from partial delamination during etching of the A‐site element, appearing as multilayer MXene particles with imperfect stacking between the layers. Frequently, full exfoliation of the structure to the single‐layer configuration is not an obvious process, or the efficiency is very low with most of the mass corresponding to multilayer particles. As commented earlier, an additional delamination step of the multilayered structure is generally required to obtain single to few‐layer MXene flakes.

Exfoliation of the MXene clays after molten salt etching often follows the procedures developed for delamination after HF etching. Thus, similar to what has been demonstrated for MXenes derived by HF etching,^[^ ^65^ ^]^ also for molten salt etching, delamination can be achieved by intercalation of an organic molecule into the interlayer space, followed by ultrasonication.^[^ ^13^, ^66^ ^]^ DMSO is one of the favorite intercalant agents, which can be combined with sonication in N‐methylformamide (NMF).^[^ ^48^, ^67^ ^]^ Isopropylamine and LiF have also been used as intercalant agents.^[^ ^46^, ^68^ ^]^ However, removal of these organic agents from the exfoliated MXene sample is arduous. On rare occasions, only sonication in ethanol has been used to delaminate.^[^ ^48^ ^]^

**Figure 10:**
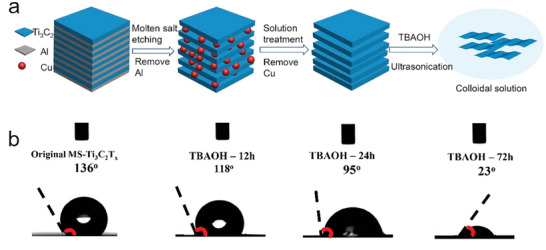
a) Illustration of the role of TBAOH as an intercalation agent favoring exfoliation of the MXene clay formed in the CuCl_2_ molten salt etching of Ti_3_AlC_2_. b) Water contact angle on an electrode made of Ti_3_C_2_ (80%), carbon black (15%), and PTFE (5%) as a function of the time in which the multilayer MXene stack has been immersed in TBAOH. Adapted with permission.^[^ ^66^ ^]^ Copyright 2021, American Chemical Society.

Simon and co‐workers demonstrated successful delamination of molten salt‐derived Ti_3_C_2_T_x_ using TBAOH and tetramethylammonium hydroxide (TMAOH) as intercalants.^[^ ^66^ ^]^ It was also noted that tetraethylammonium hydroxide (TEAOH), NaOH, and DMSO all failed to delaminate the multilayered MXene particles into single or few‐layered sheets, with the MXene retaining the accordion‐like particle morphology. Thin flakes, with thickness as small as two layers, were observed by TEM analysis of delaminated Ti_3_C_2_T_x_ samples exfoliated with TBAOH (**Figure** **10**). Preservation of the hexagonal crystal structure was further confirmed by selected area electron diffraction (SAED).^[^ ^66^ ^]^ It should be noted that this delamination was demonstrated for Ti_3_C_2_T_x_ prepared by CuCl_2_ etching of Ti_3_AlC_2_ followed by APS treatment to remove Cu metal. As previously noted, samples treated with APS contain increased O terminal groups compared to other post‐synthetic treatments, such as HCl or magnetic treatments. It remains to be determined whether or not the same delamination method may successfully be applied to MXenes with limited proportions of surface O or OH terminal groups. It appears that a higher percentage of oxygenated terminations is more appropriate for MXene exfoliation.

While the original multi‐layered MXene had a zeta potential of nearly 0 mV for a broad pH range, the TBAOH‐treated sample had a zeta potential of ≈−60 mV for pH values higher than 4, thereby forming a persistent colloidal suspension in water. Contact angle measurements showed that the previously hydrophobic surface of the MXene was modified by TBAOH treatment, increasing hydrophilicity with treatment time. The increased hydrophilicity was suggested to be the result of additional O terminations being introduced during the TBAOH intercalation and sonication. Indeed, higher proportions of oxygen for TBAOH‐treated samples were measured by EDS, as well as the undesirable formation of anatase for samples treated over 72 h, as detected by XRD.

**Figure 11:**
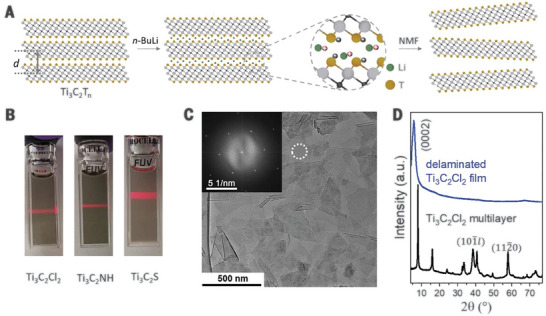
Delamination of multilayer molten salt derived Ti_3_C_2_T_z_ MXene. a) Illustration of delamination procedure with intercalation of N‐butyllithium and subsequent sonication in NMF. b) Formation of stable colloidal solutions in NMF of various Ti_3_C_2_ MXenes, showing the Tyndall effect. c) TEM micrograph of Ti_3_C_2_Cl_2_ flakes with SAED (inset) showing crystallinity with hexagonal symmetry d) Comparison of XRD spectra of multilayer MXene and glass substrate spin‐coated with delaminated MXene. Reproduced with permission.^[^ ^13^ ^]^ Copyright 2020, Science (AAAS).

Talapin and co‐workers delaminated different samples of multi‐layered Ti_3_C_2_T_x_ flakes with Cl, S, and NH surface groups by treatment with N‐butyllithium followed by bath sonication (**Figure** **11**).^[^ ^13^ ^]^ Comparison of WAXS spectra of the N‐butyllithium‐treated Ti_3_C_2_Cl_2_, before sonication, with the corresponding pristine multi‐layered sample showed an expanded interlayer spacing, suggesting the occurrence of Li^+^ intercalation before exfoliation. After sonication, size selection by centrifugation, and redispersion in NMF, a stable colloidal solution was formed with flakes exhibiting a negative zeta potential of −29.3 mV. As expected for the 2D MXene, XRD measurements only showed a broadened single (002) peak, shifted to a lower angle, indicative of separated flakes parallel to the substrate. Additionally, SAED during TEM analysis of these flakes showed the expected hexagonal pattern associated with electron irradiation along the (001) axis. Despite the high reactivity of N‐butyllithium toward acid hydrogen and chloride bonds, the original surface groups were preserved after the delamination process, as indicated by Raman spectroscopy and EDS elemental analysis. However, the high reactivity of N‐butyllithium and the strict anhydrous conditions required may make this exfoliation procedure less convenient, although it has the merit of being applicable to a wide range of termination groups.

Besides sonication, exfoliation of the accordion‐like MXene particles can also be achieved electrochemically. Repeated linear sweep voltammetry scans of Mo_2_TiC_2_T*_x_* (Mo atoms being external sandwiching the internal Ti layer) on carbon paper, over 1 000 scans, produce spontaneous exfoliation of the material accompanied by generation of Mo vacancies.^[^ ^69^ ^]^ These Mo vacancies can be useful for the preparation of single‐atom catalysts, for instance, with Pt. In this way, in a single step, exfoliation, generation of Mo vacancies and Pt single atom occupying Mo vacancies can be achieved by cycling one thousand linear sweep voltammetry scans between 0 and −0.53 V versus RHE at 20 mV s^−1^ against a Pt foil counter electrode.

## 4 MXene Surface Modification

In the first reports on MXenes, the surface groups of MXene were mostly restricted to ─F, ─OH, and O because of the prevalent highly‐aggressive fluoride‐based etching methods.^[^ ^1^, ^70^ ^]^ It is known that when HF is used as the etchant, the MXene surface is mostly dominated by F groups (Ti_2_C_2_(OH)_0.12_F_0.8_O_0.55_), while in situ HF generation methods produce MXene with O as the most prevalent surface group (Ti_2_C_2_(OH)_0.06_F_0.26_O_0.85_).^[^ ^71^ ^]^ Fluorine‐free MXenes have mainly been reported in association with the introduction of alternative MAX phase etching methods, such as NaOH etching,^[^ ^72^ ^]^ TMAOH etching,^[^ ^73^ ^]^ and electrochemical etching.^[^ ^74^ ^]^

As commented in Section 2 (on the molten salt preparation of MXenes), molten salt etching introduces other terminations different from fluorine. The study describing molten ZnCl_2_ at 550 °C as Lewis acid etchant to obtain MXene clays from the corresponding MAX phases was among the first reports on fluorine‐free MXene.^[^ ^8^ ^]^ Interestingly, ZnCl_2_ melt has been found to serve to produce first the Zn MAX, for example, Ti_3_ZnC_2_ from Ti_3_AlC_2_, from Al MAX phases by post‐synthetic replacement of Al by Zn and, subsequently, the corresponding MXene clays such as Ti_3_C_2_T_x_, along with Zn nanoparticles as a by‐product. Upon removal of the Zn metal by HCl treatment, the resulting Ti_3_C_2_T_x_ contains a high proportion of Cl surface groups, as supported by EDS analysis and XPS data. First principle calculations of the structure and the electronic properties of simulated stable structures of the Cl‐terminated Ti_3_C_2_T_x_ and Ti_2_CT_x_ models match with atomically resolved HAADF STEM images of the samples prepared by ZnCl_2_ molten salt.^[^ ^64^ ^]^ Accordingly, the available data support the preparation of chloride MXenes by ZnCl_2_ molten salt etching, resulting in the expected stoichiometric ratio of Ti_3_C_2_Cl_2_.

The preparation of fluorine‐free MXenes using molten salts appears to be general when employing late transition metal halides (including bromides and iodides) as Lewis acid etchants with demonstration of the successful formation of different halogen‐terminated MXenes using FeCl_2_, CoCl_2_, NiCl_2_, CuCl_2_, ZnCl_2_, AgCl, CdCl_2_, and CuBr_2_, and CuI.^[^ ^35^ ^]^ The abundance of Br and I in MXenes produced with the latter two etchants was confirmed by EDS analysis during SEM imaging. For these halogen salts, the importance of the redox chemistry in the etching reaction was recognized as key to achieving the successful oxidation of the A‐site element of the MAX phase by the metal cation of the salt etchant. It was thus demonstrated that the selection of a metal cation for the etchant salt with a redox potential higher than that of the A‐site element is a crucial prerequisite for the formation of MXene from the corresponding MAX phase. This procedure has considerable versatility in introducing various surface terminations.

**Figure 12:**
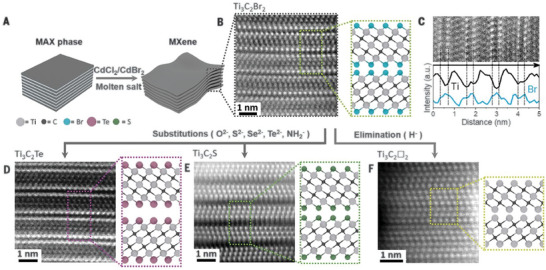
Process of molten halide salt synthesis of MXene and subsequent surface substitution in inorganic eutectic salt. a) Illustration of MAX phase etching in Lewis acid molten salt. b) Atomic‐resolution HAADF image of Ti_3_C_2_Br_2_ MXene. c) EDS line scan analysis of Ti_3_C_2_Br_2_. d,e) Atomic‐resolution HAADF images of Ti_3_C_2_Te_,_ Ti_3_C_2_S_,_ and bare Ti_3_C_2_. Reproduced with permission.^[^ ^13^ ^]^ Copyright 2020, Science (AAAS).

Thus far, reports on the molten salt synthesis of MXenes generally introduce chlorine or bromine, rarely iodine. After the initial formation of either Cl or Br terminated MXenes by molten salt synthesis, the control of possible surface groups can be expanded further by substitution reactions of the halide group.^[^ ^13^ ^]^ This strategy was demonstrated by Talapin and co‐workers by adding the anionic species to be implanted as surface termination as lithium salt dissolved in a low‐melting‐point eutectic molten salt mixture.^[^ ^13^ ^]^ It was recognized that Cl and especially Br form comparatively weak bonds with M of the MXene flake and are therefore susceptible to substitution. Compared to Cl and Br, this exchange does not occur readily for F^−^ or oxygenated surface terminal groups due to their stronger bonds. Therefore, the MXene samples obtained from the Lewis acid molten salt method are ideally suited for this exchange and can be versatile intermediates in the preparation of the final MXene with the desired termination groups. MXenes with O^2−^, S^2−^, NH^2−^, Te^2−^, and Se^2−^ surface terminations were obtained in this way from Br or Cl terminated MXenes by treatment with alkali salts (Li_2_O, Li_2_S, NaNH_2_, Li_2_Te, Li_2_Se) in eutectic mixtures of CsBr/KBr/LiBr, and analogous eutectic chloride mixtures in the case of Cl‐terminated MXenes. Even surface‐free, bare MXenes were claimed to be prepared by using LiH as reagent. This process is illustrated in **Figure** **12**. Examples of this methodology were given for Ti_3_C_2_, Ti_2_C, and Nb_2_C MXene. The resulting MXene samples were well characterized in terms of their structure and surface composition. Structural parameters for the various examples were extracted by XRD pattern fitting. Atomically resolved dark‐field STEM imaging with EDS scanning provided strong evidence to locate MXene terminations in the interlayer spaces of the MXene multilayer structures. Compositional data, including those generated by EDS, XRF, and XPS, showed that a high degree of Cl or Br substitution can be achieved throughout, near the theoretical charge‐balanced stoichiometry. It can be anticipated that surface‐free MXenes should have unique physical and chemical properties and could be an intermediate material in the preparation of other surface terminations.

Besides lithium salts, surface modification can also be achieved using other alkali metal salts. In one of these examples, the surface of Ti_3_C_2_T_x_ produced via HF etching was altered, in a post‐synthesis treatment, by K_2_CO_3_ dissolved in the LiCl/KCl eutectic salt melt at 450 °C.^[^ ^75^ ^]^ The maximum c lattice parameter measured by HRTEM of the K_2_CO_3_ treated Ti_3_C_2_T_x_ matches well with that of Li_2_O treated Ti_3_C_2_T_x_ observed from XRD patterns, suggesting that the same halide substitution for O has occurred using K_2_CO_3_ as reagent.^[^ ^13^, ^75^ ^]^ A significant reduction of the F content in favor of O is observed in TEM‐EDS and XPS data, with the latter indicating a notable increase in the contribution of the CTiO_x_ and TiO components to the O 1s peak. However, quantitative elemental analysis based on survey XPS indicates that despite the harsh treatment, the F:Ti ratio could be reduced only from 0.88 to 0.53, perhaps due to the relatively strong Ti─F bonds, as compared to the successful replacement achieved from Ti─Cl and Ti─Br groups.^[^ ^13^ ^]^ Oxygenated potassium anions, such as KO^2−^, KCO^3−^, and KCO_4_^2−^, are thought to intercalate and contribute to the substitution of F groups for O in the interlayer space.^[^ ^75^ ^]^ Well‐distributed K observed by SEM‐EDS analysis, together with the detection of K and C─O signals in XPS data, indicate remnants of these intercalated ions. It seems, however, that even this partial F‐by‐O replacement is sufficient to play a notable role in the performance improvement of this Ti_3_C_2_T_x_ material for charge storage in supercapacitors.

The substitution of Cl terminations of a Ti_3_C_2_Cl_x_ MXene by N was performed by Simon and co‐workers using a similar approach, where the MXene was treated with LiN_3_ dissolved in molten LiCl/KCl under an inert atmosphere at 550 °C.^[^ ^62^ ^]^ The Ti_3_C_2_Cl_x_ employed as starting material was obtained by FeCl_2_ molten salt etching of Ti_3_AlC_2_ and subsequent Fe removal by magnetic separation. As noted above, it is possible to preserve Cl termination in Ti_3_C_2_Cl_x_ MXene by magnetic removal of metallic Fe as opposed to treatment with APS, which would produce a partial O substitution (Ti_3_C_2_Cl_x_O_y_). Successful substitution of Cl by N was clearly indicated by the shift in the (002) XRD peak, often used to assess terminal group modifications. In the present case, the increase in the interlayer spacing from 1.10 to 1.34 nm for the Ti_3_C_2_Cl_x_ and N‐treated MXene, respectively, was considered strong evidence of surface group replacement. In addition, EDS data showed a decrease in the Ti:Cl ratio from 3:1.88 to 3:0.12, and a final Ti:N ratio of 0.63, indicating a high degree of Cl substitution. This exchange of Cl by N was further supported by local STEM‐EELS mapping, where it was observed that higher N signal intensities are measured at the interlayer space, alternating with the signals of Ti and C, as would be expected for surface termination groups in between Ti_3_C_2_ layers. As in the case of Cl to O replacement, Cl to N exchange also enhances the performance of Ti_3_C_2_T_z_ MXene for charge storage in supercapacitors, reaching almost the theoretical limit for electrolyte monolayer coverage.

Alternatively to Cl^−^ and Br^−^ substitution, an exhaustive study has shown that during the molten salt etching of the MAX phase it is directly possible to install unconventional surface terminations, such as Se, Te, Sb, P, and others, by applying the hard‐soft acid‐base principle.^[^ ^36^ ^]^ The process starts from the MAX phase and it consists in using Lewis acid etchants in molten salt. Through redox‐assisted removal of the A element, the M element can accept lone electron pairs of the anions present of the molten salt. The hard‐soft acid‐base principle guides on the stability of M─T bond. In this way, CuS or FeS dissolved into chloride fluxes render Ti_2_CS_x_ and Ti_3_C_2_S_0.5_Cl_0.5_ in the etching of the corresponding Ti MAX phases.^[^ ^36^ ^]^ In another example, Nb_2_AlC forms the accordion‐like Nb_2_CTe_x_ adding CuI and Te to the chloride melt during etching of Nb_2_AlC.^[^ ^36^ ^]^ This direct implantation of the T surface termination can avoid the need of post‐synthetic surface termination exchange.

**Figure 13:**
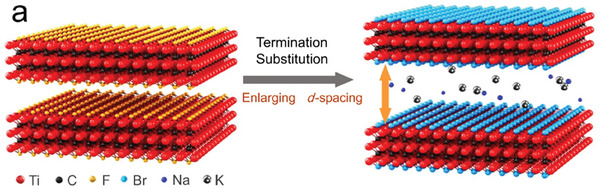
Illustration of the Lewis basic halide molten salt method using less than 50 mol% of AlX_3_ with respect to the eutectic NaX/KX mixture to achieve simultaneous surface termination exchange and interlayer distance expansion in MXene samples prepared by HF etching of the MAX phase. Adapted with permission.^[^ ^76^ ^]^ Copyright 2022, Springer Nature.

The use of molten salts has also been applied to simultaneously introduce surface functional groups and expansion of the interlayer distance after conventional HF exfoliation.^[^ ^76^ ^]^ This interlayer expansion makes it easier subsequent exfoliation to reach the monolayer state, avoiding the use of intercalating organic molecules that are later difficult to remove or the use of alkali or ammonium bases that can damage the MXene structure.^[^ ^76^ ^]^ The success of these double transformations of MXenes, i.e., interlayer distance expansion and wanted surface group termination implantation, stems from the use of Lewis basic Al halide molten salts. Eutectic mixtures of Na^+^ and K^+^ bromides or iodides in combination with the corresponding Al trihalide were used in the process, starting from the MXene sample obtained by HF etching of the corresponding MAX phase.^[^ ^76^ ^]^ When the proportion of NaX/KX (X: Br or I) in the eutectic ratio is higher than 50% with respect to the corresponding AlX_3_, then naked alkali metal cations and X^−^ are present in the molten salt. In comparison to the acidic molten salt containing higher than 50 mol% of AlX_3_, the use of less than 50% AlX_3_ results in basic melts due to the excess of halide. The presence of naked halide enables the substitution of the surface termination generated in the HF etching by halide, while the alkali metal cations intercalate within the interlayer space expanding the distance. The process is illustrated in **Figure** **13**. As a result of alkali metal intercalation, an expansion of the layer distance from 1.12 nm to 1.47 or 1.44 nm in the case of Ti_3_C_2_ samples treated with AlBr_3_/NaBr/KBr or AlI_3_/NaI/KI, respectively, was observed. In both cases, the composition of the melt was AlX_3_/NaX/KX in a molar proportion of 48.7/15.2/36.1, corresponding to the eutectic composition. This simultaneous terminal group exchange and interlayer distance increase, due to basic melts with an excess of NaX/KX in comparison to AlX_3,_ was proved to be also valid to expand and functionalize other MXenes, such as Nb_4_C_3_ and Mo_2_Ti_2_C_3_. In the latter, the Mo layers are the most external in the sheet and those becoming functionalized by Br, according to XPS.^[^ ^76^ ^]^ It would be even more convenient if the HF‐etching step could be avoided and the process could be applied directly to the MAX phase.

## 5 Molten Salt Exfoliation of Uncommon MAX Phases

The typical MAX phases used in the synthesis of MXenes have Al as the A metal, which can be removed efficiently by fluoride etching. In contrast, when the MAX phase has a metal different from Al, such as Si, Zn, or Ga, fluoride etching protocols are not so efficient, since they can decompose the MXene. Molten salt etching appears to be, in these cases, the synthesis procedure of choice since it has been validated for the synthesis of various MXenes starting from unconventional MAX‐phase precursors with A elements such as Si, Zn, and Ga.^[^ ^35^ ^]^ Specifically, molten salt has been used to prepare Ti_3_C_2_ from Ti_3_SiC_2_ by treatment with CuCl_2_ molten salt. This method enables the production of new 2D materials that are difficult or even impossible to prepare via HF etching. This synthetic route expands the range of MAX‐phase precursors that can be used and offers important opportunities for tuning the surface chemistry and properties of MXenes. The diversity and green chemistry of Lewis acids in inorganic salts offer an unexplored parameter space to optimize such etching methodology. At the same time, this approach broadens the choice of MAX‐phase families for MXene fabrication and offers opportunities to tune the surface chemistry of MXene materials by using various molten salts based on other anions (such as Br^−^, I^−^, SO_4_^2−^, or NO_3_^−^). The control of the surface termination is important to increase Li^+^ adsorption/desorption, which is required in Li^+^‐ion batteries, and also for a good blending with conductive carbon black, which is the additive commonly employed in the fabrication of battery electrodes.^[^ ^35^ ^]^

Similarly to the prevalence of Al as the A‐site element in the MAX phase, the majority of research on the synthesis of MXenes refer to metal carbides, with reports on metal nitride precursors being few and far between.^[^ ^2^, ^77^ ^]^ It appears that fluoride etching of the MAX phase does not work well for MAX nitrides, except for Ti_3_N_2_, due to their lower chemical stability. In this regard, a pioneering study on the synthesis of MXene nitrides reported the preparation using the molten salt of 2D Ti_4_N_3_.^[^ ^41^ ^]^ The reason why etching in MAX nitrides is more difficult than in the analogous carbides could be attributed to two factors. On the one hand, the calculated cohesive energies of Ti_n+1_N_n_ are less than those of Ti_n+1_C_n_. On the other hand, the formation energies of Ti_n+1_N_n_ from Ti_n+1_AlN_n_ are higher than those of Ti_n+1_C_n_ from Ti_n+1_AlC_n_. Lower cohesive energy implies lower stability of the T_in+1_N_n_ structure, whereas the higher formation energy of Ti_n+1_N_n_ implies that the Al atoms are bonded more strongly in the Ti_n+1_AlN_n_ phase, requiring more energy for their extraction. These two factors make nitride MXenes easier to dissolve in aqueous HF solution. All these issues are apparently overcome by using molten fluoride salts to etch Al from a Ti_4_AlN_3_ powder precursor. In short, Ti_3_AlN_3_ powder was mixed with a fluoride salt mixture in a 1:1 mass ratio. The fluoride salt mixture comprised 59 wt.% of KF, 29 wt.% of LiF, and 12 wt.% of NaF, corresponding to the ternary eutectic composition in this salt system. The mixture of Ti_4_AlN_3_ and fluoride salts was heated to 550 °C for 30 min, with a heating rate of 10 °C min^−1^ from room temperature under Ar flow in an alumina crucible. They further delaminate the resulting MXene with TBAOH to produce few‐layered nanosheets and monolayers of Ti_4_N_3_T_x_ (T: F, O, or OH). XRD, Raman spectroscopy, and TEM confirmed the selective etching of Al atoms from Ti_4_AlN_3_ and the preservation of Ti─N bonds in a layered hexagonal Ti_4_N_3_ structure as a result of this procedure. The method herein reported has the potential to be applied to other MAX phases to produce their corresponding MXenes.

DFT calculations of simulated Ti_4_N_3_T_x_ determined that ─O‐ terminations on Ti_4_N_3_T_x_ (T:O) are energetically the most stable termination compared to F and OH functional groups, while the OH termination is the least favorable. Relative to these simulations on the influence of Ti_4_N_3_T_x_ termination, bare, non‐terminated Ti_4_N_3_ is calculated to have the highest density of states at the Fermi level, as well as a magnetic moment of 7.0 µB per unit cell.

## 6 Molten Salt MXene Derived Materials

The previous sections have shown that inorganic molten salt etching can produce either directly in the etching step or in post‐synthetic treatments MXene samples in a wide selection of surface termination groups and even bare surface MXenes (no surface termination). This has resulted in samples with previously unattainable versatility. Surface functionalization is expected to affect nearly every MXene property by altering structural and electronic states.^[^ ^78^, ^79^, ^80^ ^]^ For example, the surface groups control interatomic distances in the MXene lattice, with telluride terminations showing a giant in‐plane lattice expansion compared with the unstrained titanium carbide lattice.^[^ ^13^ ^]^

**Figure 14:**
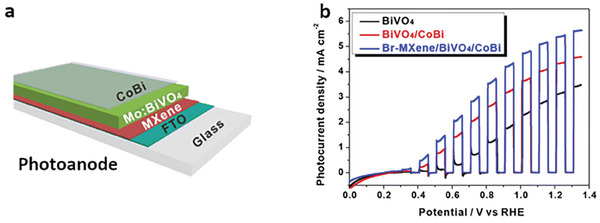
a) Structure of an improved Mo‐doped BiVO_4_ photoanode using Br‐terminated Ti_3_C_2_ as electron transport layer to increase the electrical contact with FTO. b) Comparison of the photocurrent in the linear scanning voltammetry of BiVO_4_, BiVO_4_/CoBi, and Br‐MXene/BiVO_4_/CoBi photoanodes under AM 1.5G illumination showing the improvement by Ti_3_C_2_ MXene. Adapted with permission.^[^ ^67^ ^]^ Copyright 2022, The Royal Society of Chemistry.

An interesting aspect of surface functionalization is the possibility of fabricating hybrid MXene materials with enhanced performance in energy applications. In one of these examples, Mo‐doped BiVO_4_ and Ti_3_C_2_ MXene with controlled Br surface terminations, obtained by molten salt etching, exhibited an excellent interfacial contact improving the efficiency of photoelectrochemical water‐splitting as compared with control sample photoelectrodes lacking MXene (**Figure** **14**).^[^ ^67^ ^]^

**Figure 15:**
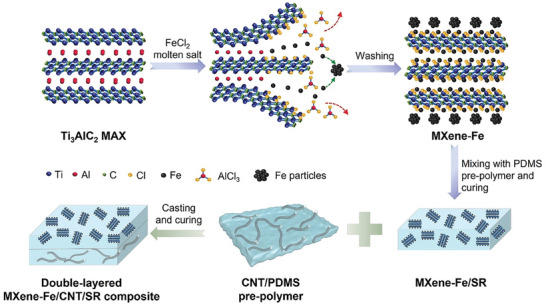
Illustration of the synthesis of double layer (upper and bottom) Ti_3_C_2_‐Fe/silicone rubber||carbon nanotube/silicone rubber composites in which the thickness of the two layers can be controlled at will. Reproduced with permission.^[^ ^81^ ^]^ Copyright 2022, Elsevier.

Double layer Ti_3_C_2_‐Fe/silicone rubber||carbon nanotube/silicone rubber composites were prepared by FeCl_2_ molten salt etching of Ti_3_AlC_2_ and using the resulting Ti_3_C_2_‐Fe MXene as filler of polydimethylsiloxane rubber (PDMS) precursors.^[^ ^81^ ^]^ The resulting PDMS with embedded MXene was mixed with another layer of carbon nanotube and PDMS precursors to form the final composites.^[^ ^81^ ^]^ **Figure** **15** illustrates the composite fabrication process. These Ti_3_C_2_‐Fe/silicone rubber||carbon nanotube/silicone rubber composites exhibit excellent tunable microwave absorption capacity for both the Ku and X‐band with potential application in aerospace and flexible electronics.

As commented earlier, molten salt etching of the A‐site metal gives rise as the primary material a MXene clay that generally contains the metal of the Lewis etching salt. In a certain way, they can be considered to be MXene derived materials that are interesting per se and can be used directly as active components in batteries, supercapacitors, and other electrical or magnetic devices. The particle size of the incorporated metal can range from a few nanometers to small clusters or even metal single‐atoms deposited on MXenes.

In one of the first examples, a Ti_3_C_2_Tx MXene/Sn material was formed in situ directly from Ti_3_AlC_2_ MAX phase precursor through the one‐step SnCl_2_ molten salt reaction at temperatures below 700 °C.^[^ ^48^ ^]^ By electron microscopy analysis, it was realized that Sn^2+^ in SnCl_2_ not only acts as Lewis acid etchant of Al in Ti_3_AlC_2_, but it is also reduced to fine metallic Sn nanoparticles that remain confined between the interlayers, expanding their distance in the resulting Ti_3_C_2_T_x_ MXene (1.15 nm) in comparison to the values expected from HF etching (0.96‐0.98 nm). Besides the nanoparticles generated in the molten salt process, MXenes can also mix with other metal particles. In one example, Ti_3_C_2_Cl_2_ prepared by molten salt etching was mixed by ball milling with Mg powder, the resulting composite exhibiting 5.4 wt.% H_2_ storage capacity, with H_2_ adsorption onset at room temperature and H_2_ desorption onset at 200 °C.^[^ ^82^ ^]^

**Figure 16:**
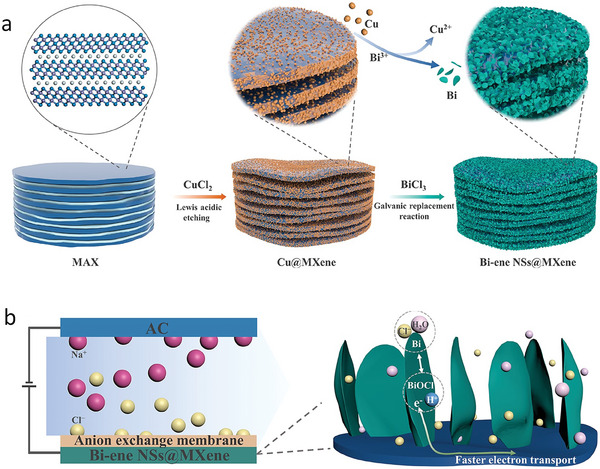
a) Synthesis of vertically aligned bismuthene nanosheet/Ti_3_CCl_2_ material by galvanic Cu by Bi replacement. b) Concept of capacitive desalination. Adapted with permission.^[^ ^83^ ^]^ Copyright 2023, American Chemical Society.

A galvanic replacement reaction, replacing Cu nanoparticles generated in the CuCl_2_ etching of Ti_3_AlC_2_ by bismuth, gives rise to a vertically oriented bismuthene‐nanosheets/MXene structured material.^[^ ^83^ ^]^ The process is illustrated in **Figure** **16**. The vertical orientation of the bismuthene nanosheets was observed by SEM, and the bismuthene structure was confirmed by XRD.^[^ ^83^ ^]^ The resulting Bi‐nanosheets/Ti_3_C_2_ exhibits interesting properties for electrochemical desalination, reaching a NaCl removal of 88.2 mg g^−1^ at 1.2 V and a fast rate and long‐term durability.

Besides fine metal nanoparticles, molten salt etching can also install clusters on the resulting MXene clay. In one of the pioneering examples, the existence of metal atoms and their interactions with MXene sheets via surface O atoms after the molten salt synthesis of Ti_3_C_2_‐Cu/Co hybrids was elucidated by XAFS.^[^ ^84^ ^]^

Using a more sophisticated methodology, there are quite a number of papers that have reported the preparation of isolated single transition metal atoms installed on the MXene layers, including Co, Cu, Ni, Fe, and others. In one example, Co atoms on Ti_3_C_2_ (Ti_3_C_2_T_x_:Co) were successfully synthesized by etching Ti_3_AlC_2_ in CoCl_2_ molten salt at 750 °C.^[^ ^85^ ^]^ The reaction of Ti_3_AlC_2_ with CoCl_2_ at elevated temperature produced initially Co nanoparticles on Ti_3_C_2_ that were subsequently treated with H_2_SO_4_ to remove the excess Co atoms loosely attached on the surface of Ti_3_C_2_, which also led to the generation of O‐based surface groups. The experimental data indicate that Co atoms were mainly aggregated in the interlayer space, while a small proportion of Co atoms was incorporated into the MXene lattice, replacing the Al atoms.^[^ ^85^ ^]^ Besides carbides, MXene nitrides, such as Ti_2_N, can also anchor single atoms. For example, starting with Ti_2_AlN MAX precursor and using the molten salt method with CoCl_2_ as etchant is suitable for etching MAX nitrides while anchoring Co single atom in vacancies formed in the MAX phase.^[^ ^60^ ^]^ Similarly, Cu single‐atoms have been immobilized on MXene layers via selective Al etching of quaternary MAX phases (Ti_3_(Al_1‐x_Cu_x_)C_2_) having bimetallic Al and Cu as the A layer.^[^ ^86^ ^]^ After selective Al etching of A layers, Cu was preserved and simultaneously immobilized onto MXene (Ti_3_C_2_Cl_x_) as single atoms. Applied as an electrocatalyst for CO_2_ reduction, the resulting material exhibited a selectivity toward methanol up to 59.1% at 1.4 V versus RHE at a rate of ≈20 mA cm^−2^, further shown to be stable for more than 30 h.^[^ ^86^ ^]^

Also, a recent paper describes the synthesis of a catalyst with Cu single‐atoms on MXene (Ti_3_C_2_T_x_) obtained by molten salt etching that is active for the 100% selective decomposition of peroxymonosulfate to singlet oxygen.^[^ ^61^ ^]^ Herein, the optimal Ti_3_AlC_2_:CuCl_2_ proportion to achieve complete Al etching was determined. It was concluded that 1:3 proportion is the optimal molar ratio since lower proportions of CuCl_2_ are not able to completely etch the Al, while higher proportions give rise to big Cu particles. Under the optimal stoichiometry and after the removal of unstable bulk Cu particles, a homogeneous distribution of Cu single‐atoms in the layers of MXene was observed by aberration‐corrected high‐angle annular dark‐field scanning TEM.^[^ ^61^ ^]^ Analysis of the images shows that Cu atoms are located at the Ti defects of the structure that are proposed to be the most stable sites. Ti vacancies are generated due to the harsh conditions of CuCl_2_ etching at high temperatures.^[^ ^61^ ^]^

As frequently commented, the metal nanoparticles formed in the molten salt etching process are removed from the MXene using an acid or oxidizing reagent. A deep study of some of the samples submitted to this leaching process has revealed the formation of single‐atom materials.^[^ ^60^, ^61^ ^]^ A totally different approach is the transformation of the metal nanoparticles into other derivatives, resulting in MXene‐derived material. In one interesting example, Huang and coworkers have vulcanized the metal nanoparticles to form metal sulfide nanoparticles supported on MXene.^[^ ^87^, ^88^ ^]^ The process seems to be simple, safe, and universal, and consists of the direct sulfurization of MXene/metal samples forming the corresponding Ti_3_C_2_T_x_/MS_γ_ heterostructures (T_x_ = ─O and ─Cl; M = Fe, Co, and Ni), where MS_γ_ nanoparticles (NPs) are grown in situ and anchored on the Ti_3_C_2_T_x_ surface via Ti─O─M interfacial bonds. Instead of eliminating various metal particles among MXene substrate after molten salt etching, this general method offers the advantage of the full utilization of molten salt etching products, increasing atom efficiency as well as greatly improving experimental safety. Additionally, the whole preparation process is not required to be carried out in water or other solvents, which greatly alleviates the occurrence of undesirable concomitant oxidation of MXenes.

## 7 Applications of Molten Salt Derived MXene Materials

After having described the state of the art in molten salt etching of the MAX phase, including unconventional MAX precursors, the use of molten salt in post‐synthetic treatments, and the preparation of MXene‐containing materials, this section intends to show the advantages in terms of performance in several applications of the MXene samples prepared by molten salt etching in comparison to etching using fluorinated reagents.

Thus far, the majority of competitive applications demonstrated for molten salt‐derived MXene materials are in electrochemical energy storage, in the form of both batteries (typically Li‐ion), and supercapacitors, and electrocatalysis. Comparatively few studies have shown successful applications in thermal catalysis,^[^ ^89^ ^]^ and as superconducting materials. Notably, with only one report detected on photoelectrocatalysis,^[^ ^90^ ^]^ and one on electromagnetic interference (EMI) shielding,^[^ ^81^ ^]^ these applications are still very scarce for molten salt‐derived MXene, despite the successes of MXene etched using fluorinated reagents in these fields.^[^ ^91^ ^]^

### 7.1 Batteries

**Figure 17:**
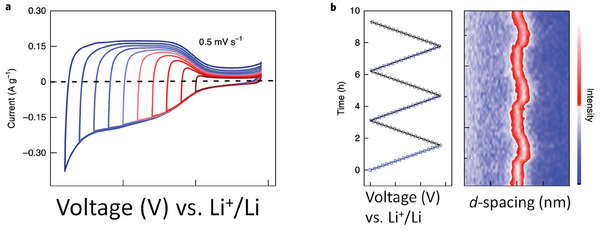
a) CV at a 0.5 mV s^−1^ scan rate with various cut‐off negative potentials. b) In situ XRD fluctuation of the (002) peak corresponding to the interlayer spacing during three consecutive anodic and cathodic scans. Reproduced with permission. Adapted with permission.^[^ ^35^ ^]^ Copyright 2020, Springer Nature.

Due to the electrical conductivity of MXene and the ability to insert Li^+^ and Na^+^ ions in the interlayer spaces of the accordion‐like form of MXenes, these materials show great promise for their use in Li^+^‐ion and Na^+^‐ion batteries, among others.^[^ ^92^, ^93^ ^]^ Considering the economic importance and expected growth in battery production and use, this application is possibly among the most important ones using MXenes. One of the main problems in ion insertion is to open space in the interlayer space to allow easy ion diffusion.^[^ ^94^ ^]^ The generation of metal particles in the molten salt MXene etching in the interlayer space is, therefore, very appropriate for producing MXene samples ready to be used in batteries. By the molten salt etching procedure, the surface terminations can be controlled, and even the interlayer spacing by intercalating the desired alkali metal cation.^[^ ^76^ ^]^ In situ XRD during cyclic voltammetry (CV) has allowed monitoring of the interlayer spacing of molten salt‐Ti_3_C_2_. In one case, following Li^+^ ion intercalation/deintercalation, an almost constant *d* spacing of ≈1.102 nm with only a variation of 0.025 nm was found. This small variation indicates the diffusion of desolvated Li^+^ ions, while blocking the co‐intercalation of the solvent and resulting in an improved electrochemical potential.^[^ ^35^ ^]^ **Figure** **17** illustrates the results of the in situ XRD. Up to 0.4 Li^+^ ions can be inserted per Ti atom, which is a remarkable charge storage capacity.^[^ ^35^ ^]^

In other reports on Li^+^ intercalation within the interlayer spaces, it has been proposed that the fine Sn nanoparticles generated in the SnCl_2_ etching of Ti_3_AlC_2_ play a role in avoiding the layer stacking and increasing the Li^+^‐ion storage capacity.^[^ ^48^ ^]^ It was proposed that Sn nanoparticles act as pillars confined between the layers of MXene, preventing the re‐stacking of MXene nanosheets and unlocking the extra interlayer spaces for Li^+^‐storage. Thanks to the in situ synthesis strategy, the large interfacial contact between Ti_3_C_2_T_x_ MXene nanosheets and fine Sn nanoparticles facilitates the diffusion of Li^+^ ions through the interlayer. It remains to be seen what the influence of the presence of these Sn metallic nanoparticles could be in the long‐term cycling stability of the electrode.

The fact that raw molten salt MXene samples can be used directly to form electrode films without any other pretreatment is a considerable advantage in comparison with other alternative etching methods to obtain MXenes from the MAX phase. However, it has been observed that, in the case of molten salt‐etched MXene electrodes, exfoliation considerably increases the performance of these samples for Li^+^‐ion batteries. In this way, films of Ti_3_C_2_Cl_2_ prepared from Ti_3_AlC_2_ by CuCl_2_ etching at 680 °C and subsequent exfoliation by sonication in the presence of TBAOH as an intercalating agent have been reported to achieve specific capacities of 225 mAh g^−1^ at a 1C rate together with an excellent rate capability of 95 mAh g^−1^ at 167 C.^[^ ^95^ ^]^ The improved power capability was ascribed to the higher electrochemically active surface area, as well as a higher surface O content and hydrophilicity, all having been introduced by the TBAOH exfoliation process.

Li^+^ storage in 1 m LiPF_6_ (in 1:1 v/v ethylene carbonate/dimethyl carbonate, (EC/DMC) was tested for Ti_3_C_2_T_x_ synthesized by etching in NiCl_2_/NaCl/KCl.^[^ ^63^ ^]^ The excess Ni metal removal was performed using various treatments, including magnetic separation in H_2_O, and by Ni dissolution in H_2_SO_4_, NaOH, and APS (all 0.5 m). Different treatments for metal removal had a significant effect on the specific Li^+^ storage capacities (measured by CV at 0.5 mV s^−1^) with 103, 109, 53, and 161 mAh g^−1^ measured for magnetic separation, H_2_SO_4_, NaOH, and APS treated Ti_3_C_2_T_x_, respectively.^[^ ^63^ ^]^ The Ti_3_C_2_T_x_‐APS sample had good capacity retention at high charge rates with capacities of 204 mAh g^1^ at a current density of 0.05 A g^−1^ and 85 mAh g^−1^ at 2 A g^−1^, as calculated from galvanostatic charge/discharge (GCD) curves. The high performance of Ti_3_C_2_T_x_‐APS was attributed to a high degree of O functionalization introduced by the APS treatment.^[^ ^63^ ^]^ Similar conclusions about the introduction of O surface termination, resulting in enhanced Li^+^ ion storage capacity have also been reported by other groups.^[^ ^96^ ^]^

The optimum temperature for the synthesis of Nb_2_CT_x_ using CuCl_2_/NaCl/KCl (with post‐synthesis APS washing) was investigated and found to be near 750 °C.^[^ ^97^ ^]^ By XRD analysis, the formation of Nb_2_CuC MAX by A‐site substitution is seen at lower temperatures (≤700 °C), while Nb carbide (NbC_x_) impurities are formed at higher temperatures (≥800 °C). At the optimal temperature of 750 °C, the typical accordion‐like structure is seen by SEM, with simultaneous EDS analysis revealing the presence of Nb, C, Cl, O, Cu, and trace amounts of Al, all shown to be well distributed throughout the structure, including the interlayer regions. This multilayered Nb_2_CT_x_ was tested as a Li^+^‐ion battery anode. GCD cycles also showed an optimum performance at a synthesis temperature of 750 °C, correlating the presence of the pure MXene phase with higher charge capacity. Consistent with other molten‐salt derived Li^+^‐ion electrodes,^[^ ^98^ ^]^ the majority of charge is introduced below 2 V versus Li^+^/Li. Comparing capacity at different rates, Nb_2_CT_x_ performs better than previously reported for molten salt derived Ti_3_C_2_T_x_.^[^ ^35^ ^]^ The maximum Li^+^ capacity of 330 mAh g^−1^ corresponds to 2.8 Li^+^ ions per Nb_2_CT_x_ unit, higher than the theoretical maximum. This excess of Li^+^ ions with respect to the stoichiometric formula is suggested to be due to the carbon black electrode additive.^[^ ^97^ ^]^ Using the Trasatti plot method to discriminate between the various contributions, it was found that the capacity has components from fast surface capacity plus diffusion‐limited capacity in the ratio of ≈1:2.5. During cycling tests at 1 A g^−1^, it was observed that the capacity nearly doubled from 100 mAh g^−1^ over 1 000 cycles. Such an increase in electrochemical performance has been seen before for MXenes,^[^ ^69^ ^]^ and is, in this case, also attributed to the increase in electrochemically active surface area due to electrochemical exfoliation.^[^ ^97^ ^]^

A Ti_2_N MXene with accordion‐like morphology was synthesized by CuCl_2_ etching of Ti_2_AlN at a relatively low temperature of 450 °C, followed by APS washing.^[^ ^98^ ^]^ A large specific surface area of 51.07 m^2^ g^−1^ was obtained for this material, which may indicate that interlayer spaces could be accessible to ions in the charge/discharge cycles, this accessibility being favorable for high charge capacity in the material. In agreement with the higher stability of O terminations, as calculated by DFT, APS treatment resulted in a high proportion of O terminations, about O (61.14%), OH (36.63%), and Cl (2.24%) according to XPS analysis. O terminations also appear to be positive from the point of view of charge storage. Analysis of the Fourier‐transformed Ti K‐edge EXAFS spectra indicates that the Ti‐Ti coordination number decreases from 9 for the MAX phase to 3.3 in the MXene, suggesting the creation of many Ti vacancies. When tested as Li^+^ storage electrodes, the molten salt derived Ti_2_N performed significantly better than that of HCl/LiF treated Ti_2_AlN. Electrochemical tests, using 1 m LiPF_6_ in EC/DMC (1:1) electrolyte, showed a high reversible capacity of 303.4 mAh g^−1^ at 100 mA g^−1^, and a capacity at a charge rate of 1000 mA g^−1^ increasing from ≈200 to 350 mAh g^−1^ over 1200 cycles.^[^ ^98^ ^]^ The remarkable performance was ascribed to the presence of O and Cl surface terminations. Further tests showed that a Li^+^‐ion capacitor device based on this MXene delivered high energy and power densities.

Regarding the influence of MXene crystallinity, it has been predicted that a change from crystalline to amorphous structure can be an effective way to increase the performance of these materials in Li^+^‐ion battery by providing a structurally more tolerant material.^[^ ^99^ ^]^ A support to this claim has been given by observing that a Nb_2_CT treated by 12 h in APS and becoming amorphous performs better (402.5 mAh g^−1^) than an analogous highly crystalline sample Nb_2_CT sample (322 mAh g^−1^).^[^ ^42^ ^]^

In addition to crystallinity, morphology can also influence the performance of MXenes in Li^+^‐ion storage and batteries. Generally, the morphology of MXene is inherited from the MAX phase and, due to their high‐temperature synthesis, these MAX particles have a micrometric 3D morphology. In a study addressing the possibility to prepare MAX phases with 1D and 2D morphology and the influence of resulting MXene morphology as anode in Li^+^‐ion batteries, it has been found that the morphology of the MAX phase depends on the morphology of the C source.^[^ ^43^ ^]^ Accordingly, using single wall carbon nanotubes or graphene aerogels as C precursors in molten salt synthesis, Ti_3_AlC_2_ and Ti_2_AlC phases as nanoribbons (1D) or flakes (2D) were prepared.^[^ ^43^ ^]^ Molten salt etching by CuCl_2_ renders then Ti_3_C_2_T_x_ and Ti_2_CT_x_ MXenes with 1D or 2D morphology that exhibit higher specific capacities in anodic scans than the corresponding analogs with conventional 3D morphology. Thus the nanosized multi‐layered Ti_2_CT_x_ with nanoribbon shape has a specific capacity value at 100 mV s^−1^ scan rate corresponding to a complete delithiation of 104 mAh g^−1^ that is higher than 74 mAh g^−1^ measured for a conventional Ti_2_CT_x_ sample.^[^ ^43^ ^]^

Besides Li^+^‐ion batteries, MXenes have also been applied to other battery types. Molten salt MXene samples have been used in Na^+^ batteries. In one report, the CuCl_2_ molten salt Ti_3_C_2_T_x_ MXene was further modified by sulfurization, obtaining a Ti_3_C_2_T_x_/CuS material.^[^ ^87^ ^]^ Ti_3_C_2_T_x_/CuS exhibits a remarkable anodic charge capacity of 347.0 mAh g^−1^, stable after 800 cycles at 3 A g^−1^, in a full Na^+^‐ion battery using Ti_3_C_2_T_x_/CuS anode with the Na_3_V_2_(PO_4_)_3_ as cathode using 1 m sodium hexafluorophosphate (NaPF_6_) dissolved in diethylene glycol materials prepared using the same procedure for anode materials for Na^+^‐ion batteries.^[^ ^88^ ^]^ Ti_3_C_2_T*_x_*/FeS_2_ heterostructure was the best‐performing anode material for a Na^+^‐ion battery, demonstrating a notable charge storage of 456.6 mAh g^−1^ at 10 A g^−1^ and long‐term cyclic stability reaching 474.9 mAh g^−1^ after 600 cycles.^[^ ^88^ ^]^ Impressively, a full sodium‐ion battery with Ti_3_C_2_T*_x_*/FeS_2_ anode delivers an excellent reversible capacity of 431.6 mAh g^−1^ after 1 000 cycles at 3 A g^−1^.^[^ ^88^ ^]^

MXenes have also been applied to Zn^2+^‐ion batteries.^[^ ^44^ ^]^ By selecting the appropriate molten salt halide, including binary and ternary mixtures, it has been possible to determine experimentally the importance of the surface termination on the performance of Ti_3_C_2_ MXene in Zn^2+^ batteries. It was found that Ti_3_C_2_I_2_ is the sample with the highest charge storage capacity reaching a value of 135 mAh g^−1^.^[^ ^44^ ^]^ Ti_3_C_2_Br_2_ obtained by CuBr_2_ molten salt etching Ti_3_C_2_ also exhibits improved performance as cathodes in Zn^2+^‐ion batteries compared to Cl^−^ terminated and the traditional HF etched MXene.^[^ ^44^ ^]^

Zn batteries operate in aqueous electrolytes, but they suffer from poor recyclability due to the formation of dendritic growth. Coating of Zn anodes with molten salt MXenes introduces hydrophobicity, aiding in the suppression of side reactions, and a high affinity for zinc, resulting in an even and homogeneous Zn deposition from the electrolyte, improving cyclability.^[^ ^100^ ^]^

### 7.2 Capacitors

Supercapacitors are electrical energy storage devices alternative to batteries.^[^ ^101^, ^102^, ^103^, ^104^ ^]^ The process is based on charge/discharge cycles corresponding to the formation/release of an electrolyte double layer on the surface of the polarized conductive electrode. While batteries rely on reversible redox processes, simple supercapacitors only require, in principle, a high electrochemical surface area that becomes polarized to immobilize ions on it by coulombic forces. Compared to batteries, supercapacitors generally enjoy high power delivery and a larger number of charge/discharge cycles (easily above 10 000).

Electrically conductive MXenes are particularly suited active materials for supercapacitors, and this has been a very active area of research since the first reports on MXenes.^[^ ^105^, ^106^, ^107^ ^]^ The use of molten salt etching in MXene synthesis has considerable advantages compared to HF etching, since the resulting materials generally exhibit a much‐improved performance.^[^ ^50^, ^108^ ^]^ These enhanced properties derive from the introduction of suitable surface terminations (implemented in the molten salt process), interlayer expansion, and the presence of metal nanoparticles that contribute introducing pseudocapacitance to the overall capacity through non‐diffusion limited Faradaic redox activity.

In one example showing the influence of the surface termination, a marked improvement in the capacitance (323.6 F g^−1^ at 1 A g^−1^) in acid media (1 m H_2_SO_4_) of Ti_3_C_2_T_x_ treated with K_2_CO_3_ in LiCl/KCl molten salt compared to pristine HF‐etched Ti_3_C_2_T_x_ was observed.^[^ ^75^ ^]^ This higher capacitance was due to the increase in surface O groups, which provides reduced charge transfer resistance and a pseudocapacitive contribution to the capacitance in which some redox processes may occur simultaneously.^[^ ^75^ ^]^ The energy storage mechanism was studied by ex situ studies of the discharged and charged material, where the expansion and contraction, respectively, of the interlayer spacing were measured by XRD.^[^ ^75^ ^]^ Characterization data of the K_2_CO_3_ treated sample indicates that surface O groups enable the reversible electrochemical conversion of Ti_3_C_2_O_2_/Ti_3_C_2_(OH)_2_ during insertion/extraction of H^+^, as deduced from the Raman spectra and by the shift in the XPS Ti‐O binding energy observed in XPS.

The influence of surface termination in MXene performance as active material in supercapacitors has been confirmed in a large number of reports.^[^ ^105^, ^107^, ^109^ ^]^ The performance of these Cl, I, and Br functionalized Ti_3_C_2_T_x_ MXenes as proton supercapacitors was measured in 3 m H_2_SO_4_ electrolyte.^[^ ^110^ ^]^ The Ti_3_C_2_T_x_ (T:Cl, Br, I) samples were synthesized by using molten salt mixtures of CuCl_2_, CuBr_2,_ or CuI, in the NaCl/KCl eutectic mixture, with post‐synthesis APS treatment to remove Cu.^[^ ^110^ ^]^ The best‐performing material was the Cl functionalized Ti_3_C_2_T_x_ reaching a specific capacity of 102 F g^−1^ at 5 mV s^−1^. However, the ion storage process was highly diffusion‐limited and short‐lived, with the capacity rapidly decreasing with increased scan‐rate and repeated cycling. These features indicate the lack of stability of the MXene under strongly acidic conditions.

In another study, it was observed that post‐synthetic exchange of Cl by N as surface termination in Ti_3_C_2_ MXene increases exceptionally the performance of the MXene as supercapacitor electrode in sulfuric acid aqueous electrolyte, achieving a capacity (300 F g^−1^ at 2 V s^−1^) near the Langmuir monolayer limit.^[^ ^111^ ^]^ This nitrification treatment improves the hydrophilicity, an important characteristic for the accessibility of the electrolyte to the electrode material, and provides a psuedocapacitive contribution with fast surface‐controlled kinetics over a broad potential range. In comparison, both the APS and magnetically treated samples showed poor performances.^[^ ^111^ ^]^

Interlayer expansion is frequently observed as a consequence of the surface termination installed in the molten salt process. This facilitates ion diffusion, which is key to obtaining a large electrochemical surface area.^[^ ^75^ ^]^ In this way, accordion‐like MXene clays can allow interlayer access of ions necessary for producing electrodes with high capacitance.

Besides large electrically active surface area, the presence of metal nanoparticles or clusters can also be favorable for the performance of molten salt etched MXenes in supercapacitors. In this way, for instance, Ti_3_C_2_‐Cu/Co hybrids featuring low charge transfer resistance, short ion/electron pathways, and rapid electrolyte ion accessibility have been reported to exhibit a high performance as active components in supercapacitors.^[^ ^84^ ^]^ The excellent electrochemical activity has been ascribed to: i) molten salt etching synthesis utilizing F‐free reagents (considered detrimental for charge storage), ii) the presence of metallic clusters connected with MXene via the surface terminating O groups, enhancing interfacial interaction and facilitating charge transfer in the MXene‐derived material, iii) stabilization of the lamellar structure of Ti_3_C_2_ sheets by sandwiched metal clusters, facilitating facile ion/electron diffusion and exposing a large active surface, and iv) MXene protection of the metal clusters, diminishing their degradation and stabilizing them during device operation.

### 7.3 Electrocatalysis

In the context of renewable energies with a low CO_2_ footprint, green electricity from hydraulics, wind, and photovoltaics has become a viable energy source that can be deployed at the massive scale required to power all human activities. For this reason, there is renewed interest in electrochemical techniques and, particularly, in converting electricity into chemical fuels. Water electrolysis, electrochemical CO_2_ reduction, and even atmospheric pressure N_2_ reduction have been among the most intensely studied electrochemical processes to convert the excess of fluctuating electrical energy into storable, ready‐to‐use chemical fuels. Regarding power‐to‐fuels conversion, the key point is to increase efficiency, and this requires minimizing the overpotential necessary to run electrochemical reactions at high current densities. As in other types of catalysis, the most efficient electrocatalysts are transition metals, where, in many cases, noble metals like supported Pt nanoparticles and IrO_2_ are used as benchmark electrocatalysts for hydrogen evolution reaction (HER) and oxygen evolution reaction (OER), respectively. These two semi‐reactions take place at a stoichiometrically balanced rate during electrochemically driven overall water splitting. The search for highly efficient HER and OER electrocatalysts at extreme acid or basic pH values has been very intense in the last two decades. Since MXenes are mostly constituted by early transition metals, their electrocatalytic activity is limited. In this regard, molten salt etching with deposition of first‐row mid‐to‐late transition metals (Fe, Co, Ni, Cu, and Zn) is very convenient, since materials suitable as electrocatalysts may be produced in this way in a single step. Electrocatalytic activity in these MXenes may derive from fine metal nanoparticles, clusters, or single atoms.

In one of the precedents, Ti_4_N_3_T_x_ synthesized with molten fluoride salts mixture as etchant showed HER activity under acidic conditions (0.5 m H_2_SO_4_), requiring an overpotential of 300 mV at 10 mA cm^−2^.^[^ ^90^ ^]^ Electrocatalysts based on non‐noble metal nanoparticles generally do not withstand acidic conditions since they dissolve. For this reason, the development of efficient HER electrocatalysts based on Earth‐abundant metals at acid conditions is an open issue. Regarding Ti_4_N_3_T_x_ performance, the reported overpotential for a relatively small current density makes this material still unsatisfactory since the ideal electrode should operate at current density values over 500 mA cm^−1^ to have commercial applicability.

In general, bimetallic electrocatalysts exhibit better performance than the corresponding monometallic counterparts.^[^ ^112^ ^]^ This general rule could also apply in the case of MXenes, in which the chemical space of bimetallic materials remains almost unexplored. In one of the scarce precedents, the electrocatalytic activity of V_0.2_Mo_0.8_N_1.2_ prepared by a novel bottom‐up molten salt method was reported.^[^ ^113^ ^]^ HAADF‐STEM showed that the V atoms were incorporated into the MXene flake as single atoms. At the optimal V/Mo atomic ratio, V_0.2_Mo_0.8_N_1.2_ exhibits an overpotential of 158 mV at 10 mA cm^−2^ for HER in 0.5 m H_2_SO_4_ and a Tafel slope of 39 mV dec^−1^.^[^ ^113^ ^]^ These values are still worse than those of Pt/C as reference (an overpotential of 23 mV at 10 mA cm^−2^ and a Tafel slope of 29 mV dec^−1^), but better than other related materials, including MoN_1.2_ and VN*_x_*, or other V‐Mo nitride MXene compositions.^[^ ^113^ ^]^ Clearly, there is still much potential in this area to develop multi‐metallic nitride MXenes as electrocatalysts, and the molten salt etching methods appear very well suited for the preparation of these materials.

**Figure 18:**
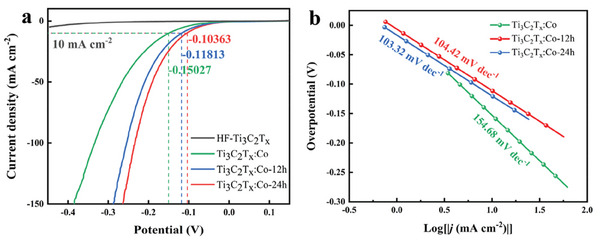
a) HER polarization curves in 1.0 m KOH electrolyte using as electrocatalysts the as prepared molten salt sample (Ti_3_C_2_T_x_:Co), after Co removal by soaking with H_2_SO_4_ for 12 (Ti_3_C_2_T_x_:Co‐12 h) or 24 h (Ti_3_C_2_T_x_:Co‐24 h) or being prepared by HF etching (HfeTi_3_C_2_T_x_). Note that the values of Ti_3_C_2_T_x_:Co‐12 h and Ti_3_C_2_T_x_:Co‐24 h in the plot have been wrongly indicated and they should be reversed according to the text. b) Tafel plots of the Ti_3_C_2_T_x_:Co, Ti_3_C_2_T_x_:Co‐12 h and Ti_3_C_2_T_x_:Co‐24 h hybrids. Adapted with permission.^[^ ^85^ ^]^ Copyright 2021, Elsevier Ltd.

A study on Ti_3_C_2_T_x_:Co MXene obtained by CoCl_2_ etching of Ti_3_AlC_2_ MAX phase at 750 °C has shown that the HER electrocatalytic activity of the material under basic conditions depends on the Co content and the way in which Co excess is removed.^[^ ^85^ ^]^ The best sample was obtained by soaking the Ti_3_C_2_T_x_:Co for 12 h in 0.5 M aqueous H_2_SO_4_, shorter or longer times removing less Co excess or causing damage to the Ti_3_C_2_T_x_ structure, resulting in poorer performance. The best Ti_3_C_2_T_x_:Co sample requires 103.6 mV overpotential to deliver a current density of 10 mA cm^−2^.^[^ ^85^ ^]^ **Figure** **18**a presents the linear sweep voltammetry, showing the remarkable influence of the presence of Co on the electrocatalytic performance. The slope of the Tafel plot (Figure 18b) was also smaller for the Ti_3_C_2_T_x_:Co sample after 12 h on H_2_SO_4_, indicating the highest kinetics was achieved with this electrode.^[^ ^85^ ^]^ In addition, the Ti_3_C_2_T_x_:Co electrode showed good stability with an increase of only a 5 mV of overpotential at 10 mA cm^−2^ after 3 000 cycles during CV. Similarly, the electrode exhibited high stability in chronoamperometry for 10 h, also at a current density of 10 mA cm^−2^.

In ather example of single‐atom electrocatalysis, Garcia and coworkers prepared Fe single atom on (Fe)Ti_3_C_2_Cl, (Fe)Ti_3_C_2_Br, and (Fe)Ti_3_C_2_(NH) showing the remarkable influence that the surface termination plays on the electrocatalytic 2e^−^ reduction of O_2_ to H_2_O_2_, the material with the highest faradic efficiency toward H_2_O_2_ being the one having NH on the external surface.^[^ ^37^ ^]^

**Figure 19:**
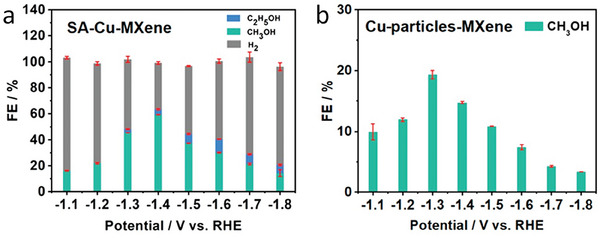
a) Product distribution as a function of the cell voltage measured for Cu single atoms on Ti MXene. Note the volcano plot for methanol production. b) Faradaic efficiency for methanol production for Cu nanoparticles supported on MXene. Adapted with permission.^[^ ^86^ ^]^ Copyright 2021, American Chemical Society.

Besides HER, CO_2_ reduction reaction (CO_2_RR) is also a key electrochemical reaction to convert power into fuels. In certain ways, CO_2_RR is more challenging than HER since the target is to get high selectivity toward desired products such as methanol and short‐chain alcohols or ethylene and C_2+_ hydrocarbons. Cu‐based electrocatalysts are widely studied due to their ability to form C_2+_ products under CO_2_RR conditions. In an early report on the application of molten salt‐derived MXene as a CO_2_RR electrocatalyst, Cu single‐atoms on Ti_3_C_2_Cl_z_ were obtained by ZnCl_2_ molten salt etching of a quaternary Ti, Cu, and Al MAX phase (Ti_3_Al_x_Cu_1‐x_C_2_).^[^ ^86^ ^]^ In the etching process, Al is selectively removed, AlCl_3_ is evaporated from the mixture, while Cu remains in the structure. These Cu single atoms on MXene exhibit high methanol selectivity, the Faradaic efficiency following a volcano plot with the polarization voltage. **Figure** **19** shows the product selectivity achieved in CO_2_RR for Cu single atoms on MXene and provides a comparison with the performance of an analogous electrode having Cu nanoparticles. As can be seen, the Faradaic efficiency for methanol of Cu single atoms is about three times higher than that of Cu nanoparticles on MXene.^[^ ^86^ ^]^ Since methanol is one of the most wanted products and it is generally difficult to form selectively, the Faradaic efficiency obtained for methanol is remarkable. However, unfortunately, the current density measured at 1.4 V, ≈20–30 mA cm^−2^ is still unsatisfactorily low for any commercial application. More effort is still necessary to develop MXene electrodes in which the methanol selectivity is maintained at high current intensities above 500 mA cm^−2^.^[^ ^114^ ^]^

### 7.4 Photoelectrocatalysis

High photocurrent intensity at low polarization potential is one of the prerequisites to develop a photoelectrochemical cell. Typically, a thin film of photoresponsive material is deposited on a conductive, transparent electrode. Indium‐tin oxide (ITO) and F‐doped tin oxide (FTO) are the two most widely used transparent electrodes for this purpose. As commented in Section 6 on MXene hybrid and composite materials, MXenes obtained from molten salt can be used as an electron transport layer to diminish the ohmic resistance between Mo‐doped BiVO_4_ and the electrode, thus, increasing the photocurrent intensity.

**Figure 20:**
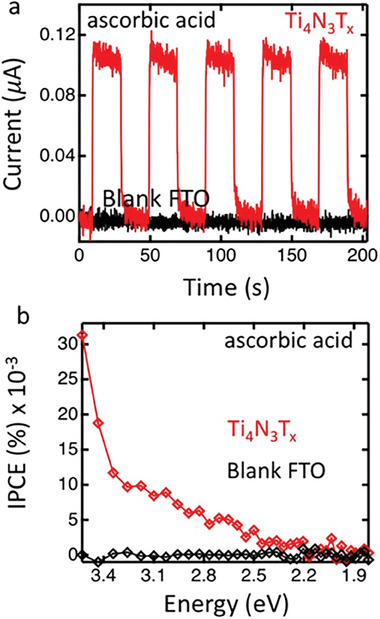
a) Transient photocurrent and b) Incident photon to current efficiency (IPCE) of a T_i4_N_3_T_x_ electrode on FTO in the presence of ascorbic acid as an electron donor. Reproduced with permission.^[^ ^90^ ^]^ Copyright 2019, American Chemical Society.

Besides charge carrier transport layers, MXenes can also be designed to be used as the photoresponsive component of a photoelectrode. In one of these examples, Ti_4_N_3_T_x_ synthesized using a eutectic molten fluoride salt mixture as etchant (LiF, NaF, KF) and TBAOH as exfoliant was used as photoelectrode.^[^ ^90^ ^]^ **Figure** **20** shows the anodic photocurrent response of the Ti_4_N_3_T_x_ obtained by fluoride molten salt synthesis on FTO under chopped monochromic illumination (530 nm wavelength) at an open circuit potential of 0.49 V versus RHE in the presence of ascorbic acid as a hole scavenger. By interpretation of the photoluminescence spectra and particularly the shift of the emission maximum to higher energies upon increasing excitation energy, it was proposed that the photocatalysis derives from defect‐states rather than from conventional band‐to‐band transitions.

### 7.5 Superconductors

Even though the electrical conductivity of MXenes can be high compared to many other materials, there is considerable interest in developing materials with the highest possible conductivity and in developing superconducting materials with transition temperatures as high as possible. Some studies report that MXenes, obtained from the molten salt etching method, can exhibit superconducting properties.

For instance, Talapin and co‐workers compared the temperature‐dependent resistivity of bare Nb_2_C and functionalized Nb_2_CT_x_ samples (T = Cl, O, S, NH, Se).^[^ ^13^ ^]^ Transitions to the superconducting state were detected for Nb_2_CCl_2_ (T_c_ ≈ 6.0 K), Nb_2_CS_2_ (T_c_ ≈ 6.4 K), Nb_2_C(NH) (T_c_ ≈ 7.1 K), and Nb_2_CSe (T_c_ ≈ 4.5 H), while no transition could be seen above 1.8 K for the parent MAX phase Nb_2_AlC, Nb_2_CO_x_, the bare Nb_2_C, or Nb_2_CT_x_ prepared by the traditional HF etching route. An initial decrease in the conductivity of Nb_2_CCl_2_ on cooling was observed, indicating its metallic behavior.

In another report, Zhang and co‐workers compared the temperature‐dependent resistivity and magnetization of Nb_2_C MXene prepared by fluoride‐based (HF and LiF/HCl) and molten salt (CdCl_2_) methods.^[^ ^115^ ^]^ The presence of F in the former and Cl in the latter was confirmed by XPS and EDX. No superconducting properties were observed for Nb_2_CF_x_, while Nb_2_CCl_x_ showed a clear transition to the superconducting state with T_c_ = 5.2 K,^[^ ^115^ ^]^ in agreement with the previous observation by Talapin and co‐workers.^[^ ^13^ ^]^ Here, however, an initial increase in the resistivity is seen upon cooling of Nb_2_CCl_x_, indicating non‐metallic behavior. The superconducting parameters of Nb_2_CCl_x_ were determined by a systematic study of the electrical and magnetic properties, which further demonstrated the material to be a type II superconductor. Analysis of experimental temperature‐dependent conductivity as well as DFT calculations found that the density of states on the Fermi surface, a feature thought to dominate when superconducting behavior occurs, is higher in Nb_2_CCl_x_ than in Nb_2_CF_x_. Considering the large diversity of compositions, surface terminal groups, and structures that can be prepared by the molten salt etching procedure, further studies on the superconducting properties of other MXene samples can be forecast.

### 7.6 Electromagnetic Interference Shielding

Considering the importance of electromagnetic waves in telecommunications, sensing, detection and their technical implication of materials able to respond to electromagnetic stimuli, there is an increasing interest in developing more efficient multifunctional materials for these applications. MXenes, having a notable electrical conductivity and having nanometric thickness are very attractive materials in this field. In fact, it has been reported that Ti_3_C_2_T_x_ films of a few microns thickness are among the most efficient electromagnetic shields for this narrow thickness with higher than 50 dB for only 2.5 µm thick films. This extraordinary shield effect has been proposed to derive from the good mechanical stability, 2D morphology, nanometric atomic thickness, and facile surface coating with MXene films, combined with their high electrical conductivity and their metal‐like character with a high reflectivity for electromagnetic waves.^[^ ^116^ ^]^ Most of the reports in this application report MXene samples that have been obtained by F‐based etching of the corresponding MAX phase.^[^ ^117^, ^118^, ^119^, ^120^ ^]^

Molten salt synthesis of MXenes offer the possibility to obtain in a single step the MXene material containing metal nanoparticles, the resulting material being very interesting for its application as an electromagnetic wave shield. In one of the examples, Fe/Ti_3_C_2_ was obtained using FeCl_2_ as Lewis acid etchant of the Ti_3_AlC_2_ phase in NaCl‐KCl molten salt at 700 °C.^[^ ^81^ ^]^ This Fe/Ti_3_C_2_ material was used as an active filler in a polydimethylsiloxane matrix. The polydimethylsiloxane was prepared from the hydrogen dimethicone and vinyl dimethicone monomers in a proportion 1:6, approximately. The resulting Fe/Ti_3_C_2_ embedded in a polydimethylsiloxane matrix was used in combination with carbon nanotubes (CNT) in the same matrix to prepare a double‐layer rubber film to absorb microwave radiation.^[^ ^81^ ^]^

**Figure 21:**
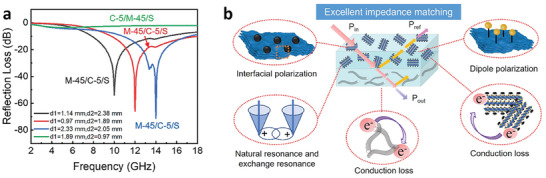
a) Frequency‐tunable reflection loss of double‐layered MXene‐Fe‐45%/CNT‐5%/Silicon‐Rubber composites, and b) scheme of the microwave absorption mechanisms. Adapted with permission.^[^ ^81^ ^]^ Copyright 2022, Elsevier.

In this double‐layer film, the role of Fe/Ti_3_C_2_ layer is to absorb the microwave radiation, while that of the CNT layer is to be a reflective layer. The combination of the two layers results in very effective radiation absorption by multiple mechanisms (**Figure** **21**b) which can be tuned by altering the thickness of the layers (Figure 21a) and the filler contents. These parameters were optimized for a minimum reflection loss of −65.9 dB with a wide bandwidth (8.24 GHz) over the Ku and X‐bands. The excellent microwave absorption of these metal‐containing MXene materials makes them very promising components in aerospace and electronics.

### 7.7 Heterogeneous Thermal Catalysis

Due to their notable electrical conductivity, MXenes have widely been used as electrocatalysts and applications related to electrical energy conversion and storage.^[^ ^121^, ^122^, ^123^ ^]^ In comparison, the use of MXenes as thermal catalysts is an almost completely unexplored field,^[^ ^38^, ^89^ ^]^ although there is a growing interest motivated by the presence of intrinsic catalytic sites on MXene structure, as for instance to promote hydroaminations.^[^ ^124^ ^]^ This general situation of the current state of the art is also reflected in the subarea regarding MXenes obtained by the molten salt etching method.

From the most recent results, it appears that MXenes obtained from molten salts may be a general way to obtain single‐atom metal catalysts in a single step from the MAX phase and subsequent washings to remove metal excess. Apparently, some defects are caused by the harsh molten salt conditions, and M‐site metal atom vacancies are replenished by the late transition metal of the molten salt. In this way, it appears that MXenes can compete as supports of single‐atom metal catalysts with carbon supports and metal oxides.^[^ ^125^ ^]^

Currently, the number of reports describing the use of MXene samples prepared by the molten salt procedure is very limited and deals solely with peroxide decomposition activation. In one example, Co_3_O_4_ nanoparticles were grown in situ in a single step on Ti_3_CCl_2_ during Ti_3_AlC_2_ etching/calcination in CoCl_2_ in eutectic LiCl/KCl mixture and used as catalysts for peroxymonosulfate activation in the degradation of ornidazole antibiotic.^[^ ^126^ ^]^ In another case, starting from Ti_2_AlN and using a 2:1 molar ratio of CoCl_2_ at 1 000 °C or CuCl_2_ at 700 °C, Al etching, generation of Ti defects, and installation of Co or Cu single atoms stabilized at the MXene defects occur in a single step, though subsequent removal of excess Co/Cu particles is required.^[^ ^60^ ^]^ The resulting Co or Cu single atoms on Ti_2‐x_N MXene exhibit high catalytic activity for the degradation of carbamazepine, sulfamethoxazole, and 2,4‐dichlorophen as model pollutants using peroxymonosulfate. Co‐SA/Ti_2‐x_N (SA meaning single atom) was more active than the Cu analog, seemingly due to the generation of Co^III^ assisted by Ti^IV^/Ti^III^ redox pair in the MXene layer. The catalytic activity of Co‐SA/Ti_2‐x_N (1.6 wt.% Co content) increases with the pH, a trend that is in general opposite to what is observed for metal salts that tend to form hydroxides and precipitate at neutral and basic pH values. Using quenchers and spin‐trapping agents, evidence in support of the generation of OH and SO_4_^−^ radicals were obtained, while the operation of singlet oxygen oxidation was ruled out.^[^ ^60^ ^]^ A colorimetric glucose sensor was recently reported based on the peroxidase catalytic activity of Ti_3_C_2_/Co nanosheets obtained from CoCl_2_ molten salt.^[^ ^127^ ^]^ Herein, Ti_3_C_2_/Co nanosheets embedded in sodium alginate hydrogel promoted tetramethylbenzidine oxidation to its deep blue oxidized form by H_2_O_2_ generated by oxidation of glucose by glucose oxidase. This system was able to detect 33 µm of H_2_O_2_ and 1.7 µm of glucose.^[^ ^127^ ^]^

In contrast to the previous generation of oxyl radicals, a recent study on Cu‐SA/Ti_3_C_2_ obtained by CuCl_2_ molten salt of Ti_3_AlC_2_ MAX phase at 900 °C and subsequent removal of Cu excess by aqueous HCl results in a material that exhibits 100% selectivity toward singlet oxygen generation from peroxymonosulfate decomposition.^[^ ^61^ ^]^ EXAFS indicates that Cu single atoms were bonded to three O atoms in its first coordination sphere, Cu‐SA/Ti_3_C_2_ exhibits an enhanced catalytic activity toward the removal of bisphenol A compared to other related samples, including Cu NPs onTi_3_C_2_ or pristine Ti_3_C_2_ as well as other benchmark catalysts.^[^ ^61^ ^]^

Given the breadth of this area, it can be easily predicted that this application of molten salt‐prepared MXenes is going to be burgeoning beyond electrocatalysis.

## 8 Conclusion and Perspectives

The present review has shown the advantages that molten salt etching of MAX phases presents in comparison to the more conventional F‐reagent etching in terms of general applicability, control of the surface terminations and the interlayer spacing, simultaneous uniform deposition of fine transition metal nanoparticles or single atoms, and scalability for multigram synthesis, among others. It has been shown that the MXene‐metal nanoparticle hybrids are frequently well‐suited for their direct application as active materials in supercapacitors, battery anodes, and electro‐/photoelectrocatalysts. In this way, in only four years since the discovery of the method, the molten salt etching of MAX precursors to obtain MXenes is rapidly gaining traction as an alternative etching procedure with many advantages over traditional fluoride methods. Molten salt etching is likely to be used extensively in the near future. Further progression is also expected in the facile synthesis of application‐specific, ready‐to‐use materials.

Further development is needed to understand, control, and overcome the weak points of the current state of the art of molten salt MAX phase etching. First, the scope of the application of the molten salt procedure is not fully established. The success in molten salt etching methods for the preparation of nitride MXenes has been noteworthy, especially considering the limited viability of fluoride‐based etching methods for nitride MXenes.^[^ ^128^ ^]^ However, the current body of work on nitride MXene is still limited compared to that of carbide MXene, leaving significant scope for further research in nitride MXene variations (for example more M compositions, and surface groups), and unexplored applications. Furthermore, we find no reports on molten salt etching of MAB phases to produce MBene.

Another open issue is the generation of defects on the MXene sheets. It is not yet fully clear how these defects are created, how to control their nature and density, and how to use them to prepare single atom materials.

Further progress on the “greenness” of the molten salt method is still necessary. This includes the use of lower temperatures and energy consumption to form the melt and perform the etching by using special mixtures and heating procedures such as microwaves or plasmas, or mechanochemical etching. In addition, transformation of the present procedures requiring large excess amounts of late transition metals into catalytic etching processes in which sub stoichiometric amounts can be employed would represent a significant advance in the field with considerable positive impact in the greenness of the process.

## Conflict of Interest

The authors declare no conflict of interest.
